# Oxysterol Sensing through the Receptor GPR183 Promotes the Lymphoid-Tissue-Inducing Function of Innate Lymphoid Cells and Colonic Inflammation

**DOI:** 10.1016/j.immuni.2017.11.020

**Published:** 2018-01-16

**Authors:** Johanna Emgård, Hana Kammoun, Bethania García-Cassani, Julie Chesné, Sara M. Parigi, Jean-Marie Jacob, Hung-Wei Cheng, Elza Evren, Srustidhar Das, Paulo Czarnewski, Natalie Sleiers, Felipe Melo-Gonzalez, Egle Kvedaraite, Mattias Svensson, Elke Scandella, Matthew R. Hepworth, Samuel Huber, Burkhard Ludewig, Lucie Peduto, Eduardo J. Villablanca, Henrique Veiga-Fernandes, João P. Pereira, Richard A. Flavell, Tim Willinger

**Affiliations:** 1 Center for Infectious Medicine, Department of Medicine, Karolinska Institutet, 141 86 Stockholm, Sweden; 2 Champalimaud Research, Champalimaud Centre for the Unknown, 1400-038 Lisboa, Portugal; 3 Immunology & Allergy Unit, Department of Medicine, Karolinska Institutet, 171 76 Stockholm, Sweden; 4 Unité Stroma, Inflammation & Tissue Repair, Institut Pasteur, 75724 Paris, France; 5 INSERM U1224, 75724 Paris, France; 6 Institute of Immunobiology, Kantonsspital St. Gallen, 9007 St. Gallen, Switzerland; 7 Manchester Collaborative Centre for Inflammation Research, University of Manchester, Manchester M13 9PL, UK; 8 I. Medizinische Klinik und Poliklinik, Universitätsklinikum Hamburg-Eppendorf, 20246 Hamburg, Germany; 9 Department of Immunobiology, Yale University School of Medicine, New Haven, CT 06520, USA; 10 Howard Hughes Medical Institute

**Keywords:** innate lymphoid cells, oxysterols, GPR183, EBI2, cell migration, colon, lymphoid tissue, inflammation

## Abstract

Group 3 innate lymphoid cells (ILC3s) sense environmental signals and are critical for tissue integrity in the intestine. Yet, which signals are sensed and what receptors control ILC3 function remain poorly understood. Here, we show that ILC3s with a lymphoid-tissue-inducer (LTi) phenotype expressed G-protein-coupled receptor 183 (GPR183) and migrated to its oxysterol ligand 7α,25-hydroxycholesterol (7α,25-OHC). In mice lacking *Gpr183* or 7α,25-OHC, ILC3s failed to localize to cryptopatches (CPs) and isolated lymphoid follicles (ILFs). *Gpr183* deficiency in ILC3s caused a defect in CP and ILF formation in the colon, but not in the small intestine. Localized oxysterol production by fibroblastic stromal cells provided an essential signal for colonic lymphoid tissue development, and inflammation-induced increased oxysterol production caused colitis through GPR183-mediated cell recruitment. Our findings show that GPR183 promotes lymphoid organ development and indicate that oxysterol-GPR183-dependent positioning within tissues controls ILC3 activity and intestinal homeostasis.

## Highlights

• ILC3s sense cholesterol metabolites (oxysterols) through the receptor GPR183
• GPR183 and its ligand 7α,25-OHC promote ILC3 migration to CPs and ILFs
• GPR183 and 7α,25-OHC are critical for CP and ILF formation in the colon
• GPR183 controls inflammatory tissue remodeling during immune-mediated colitis

## Graphical Abstract

**Figure:**
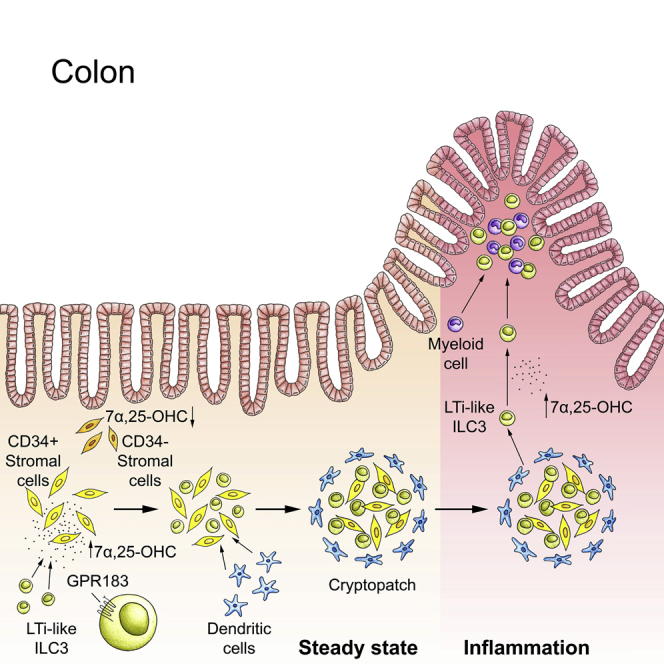

## Teaser

ILC3s maintain healthy organ function in the intestine, but how ILC3s directly detect environmental cues is poorly understood. Emgård et al. find that GPR183 and oxysterols control the localization and LTi function of ILC3s and thereby promote the formation of colonic lymphoid tissues in the steady state and inflammation.

## Introduction

Innate lymphoid cells (ILCs) are recently described immune cells of lymphoid origin and include cytotoxic natural killer (NK) cells and interleukin-7 receptor alpha (also known as CD127)^+^ subsets, which, similar to T helper (Th) lymphocytes, can be distinguished on the basis of signature transcription factors and effector cytokines: (1) ILC1s require the transcription factor T-BET and produce interferon-γ. (2) ILC2s express the transcription factor GATA3 and produce the type 2 cytokines interleukin 5 (IL-5) and IL-13. (3) ILC3s are dependent on the transcription factor RAR-related orphan receptor gamma t (RORγt) and have the ability to produce IL-17 and/or IL-22.

ILC3s are enriched in the intestine, where they maintain healthy tissue function by orchestrating lymphoid-organ development, containment of commensal bacteria, tissue repair, host defense, and regulation of adaptive immunity (Artis and Spits, 2015, Diefenbach et al., 2014, Eberl et al., 2015, McKenzie et al., 2014, Serafini et al., 2015, Sonnenberg and Artis, 2015). ILC3s can be divided into two main populations with distinct ontogeny, transcriptional programs, and localization within the gut: (1) C-C motif chemokine receptor 6 (CCR6)^−^ ILC3s co-expressing RORγt and T-BET are mainly found scattered throughout the lamina propria (Luci et al., 2009, Sanos et al., 2009, Satoh-Takayama et al., 2008). (2) CCR6^+^NKp46^−^ fetal lymphoid tissue inducer (LTi) and adult LTi-like cells expressing c-KIT (also known as CD117) seed the gut during fetal development, develop along a pathway distinct from that of other ILCs, and promote lymphoid tissue development (Eberl and Littman, 2004, Eberl et al., 2004, Mebius et al., 1997, Sawa et al., 2010). Accordingly, LTi cells reside in intestinal lymphoid structures, called cryptopatches (CPs) (Kanamori et al., 1996) and isolated lymphoid follicles (ILFs) (Hamada et al., 2002), which are collectively referred to as solitary intestinal lymphoid tissues (SILTs) (Buettner and Lochner, 2016, Randall and Mebius, 2014). CPs are clusters of LTi-like ILC3s surrounded by dendritic cells (DCs) within a network of stromal cells, whereas ILFs additionally contain B cells. CPs and ILFs develop postnatally through the activity of adult LTi-like ILC3s that produce lymphotoxin (Eberl and Littman, 2004, Kruglov et al., 2013, Tsuji et al., 2008). Whereas lymphoid organogenesis in the small intestine has been well studied, the specific factors required for SILT development in the colon, beyond lymphotoxin, are unknown.

Environmental cues, such as microbial, dietary, and neuronal signals, regulate the differentiation and function of ILC3s. However, the identities of any additional cues and the receptors that detect them remain unknown. An important class of proteins enabling cells to sense extracellular cues are G-protein-coupled receptors (GPCRs), which mediate cellular responses to diverse environmental signals. We therefore hypothesized that ILC3 function is regulated by GPCRs that recognize endogenous metabolites. In this regard, we focused on G-protein-coupled receptor 183 (GPR183, also known as EBI2), a GPCR that instructs antibody (Ab) responses in lymphoid organs through the positioning of B cells, T cells, and DCs (Gatto et al., 2009, Gatto et al., 2013, Li et al., 2016, Pereira et al., 2009, Yi and Cyster, 2013). GPR183 is a receptor for oxysterols (Hannedouche et al., 2011, Liu et al., 2011), hydroxylated metabolites of cholesterol, which have pleiotropic roles in lipid metabolism, immunity, and inflammation (Cyster et al., 2014). The most potent GPR183 ligand is 7α,25-hydroxycholesterol (7α,25-OHC). Synthesizing 7α,25-OHC from cholesterol requires the enzymes cholesterol 25-hydroxylase (CH25H) and cytochrome P450, family 7, subfamily b, polypeptide 1 (CYP7B1), whereas hydroxy-delta-5-steroid dehydrogenase, 3 beta- and steroid delta-isomerase 7 (HSD3B7) metabolizes 7α,25-OHC into bile acid precursors that lack GPR183 ligand activity. Given that GPR183 regulates immune cell migration, we reasoned that oxysterols could function as guidance cues for ILC3s.

Here, we report that ILC3s sensed oxysterols through GPR183, which was highly expressed by LTi-like ILC3s. 7α,25-OHC-synthesizing enzymes were produced by fibroblastic stromal cells found in intestinal lymphoid structures, and the GPR183 ligand 7α,25-OHC acted as a chemoattractant for ILC3s. GPR183 and 7α,25-OHC were required for ILC3 localization to lymphoid structures in the colon, and ablation of *Gpr183* in ILC3s caused a defect in the formation of colonic CPs and ILFs. The same phenotype was observed in mice lacking *Ch25h*, demonstrating a requirement for oxysterols in lymphoid tissue organogenesis. Furthermore, 7α,25-OHC was increased by inflammatory signals, and GPR183 controlled inflammatory cell recruitment during colitis. Consequently, *Gpr183*-deficient mice were less susceptible to colitis in an innate model of intestinal inflammation. Our results establish a role for a lipid ligand and its cell-surface receptor in controlling ILC3 migration, lymphoid tissue development, and inflammatory responses in the intestine.

## Results

### LTi-like ILC3s Highly Express GPR183 and Migrate toward 7α,25-OHC

**Figure 1:**
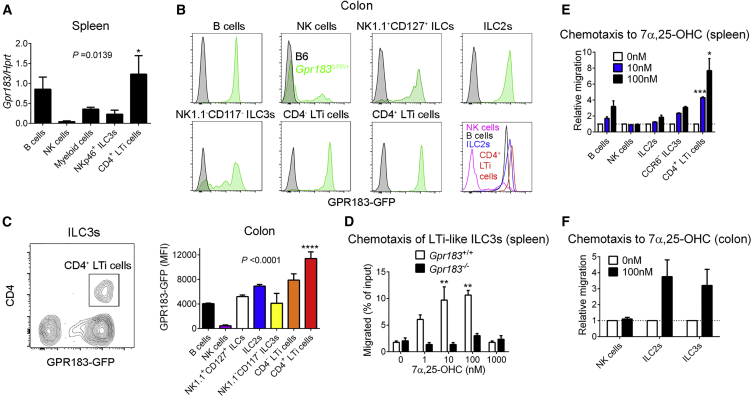
LTi-like ILC3s Highly Express GPR183 and Migrate toward 7α,25-OHC (A) *Gpr183* mRNA expression in the indicated cell populations from the spleen (n = 2–6). mRNA expression was normalized to *Hprt*. (B) GFP expression in lamina propria B cells and ILC subsets from the colon of *Gpr183*^*GFP*/*+*^ reporter mice (green histograms) and B6 control mice (gray histograms). (C) Left panel illustrates high GPR183-GFP expression in CD4^+^ LTi-like ILC3s from the colon. Right panel shows mean fluorescence intensity (MFI) of GPR183-GFP expression in the indicated cell populations from (B) (n = 6). (D–F) Transwell migration of splenic LTi-like ILC3s (Lin^−^CD90.2^+^CD127^+^NK1.1^−^) from *Rag1*-deficient *Gpr183*^+/+^ and *Gpr183*^−/−^ mice (D), splenic B cells from B6 mice and ILC subsets from *Rorc(γt)*^*GFP*^*Rag1*^−/−^ mice (E), and colonic ILC subsets from *Rag1*^−/−^ mice (F) to 7α,25-OHC (n = 2–3). Data are represented as means ± SEM. ^∗^p < 0.05, ^∗∗^p < 0.01, ^∗∗∗^p < 0.001, ^∗∗∗∗^p < 0.0001 by one-way ANOVA with Tukey’s post-test. Data are representative of or combined from two (D and E) or three (A–C and F) experiments. See also Figures S1 and S2.

It is unknown whether ILCs express GPR183 and whether the GPR183 ligand 7α,25-OHC regulates ILC function. To test our hypothesis that GPR183 controls ILC migration, we first determined *Gpr183* expression in ILC subsets. As expected, *Gpr183* mRNA was expressed in purified B cells from the spleen, but not in NK cells, whereas ILCs with an LTi phenotype (Lin^−^CD127^+^NKp46^−^CD4^+^) abundantly expressed *Gpr183* (Figure 1A). To confirm these findings, we used *Gpr183*^*GFP/+*^ reporter mice (Pereira et al., 2009) and focused on the colon, given that it has the full spectrum of ILC subsets (Figure S1). As in the spleen, NK cells largely lacked *Gpr183* mRNA, whereas other ILC types expressed *Gpr183* (Figure 1B). Among all ILC subsets, CD4^+^ LTi-like ILC3s had the highest *Gpr183* expression (Figures 1B and 1C). ILC3s from the small intestine (Figures S2A–S2C) and lymph node (Figure S2D) also expressed *Gpr183*. Moreover, mRNA for the GPR183 ligand-regulating enzymes CH25H, CYP7B1, and HSD3B7 was expressed in both the small intestine and colon (Figure S2E). The high *Gpr183* mRNA expression in LTi-like ILC3s led us to ask whether ILC3s express functional GPR183 on the cell surface. To address this question, we performed chemotaxis assays to the known GPR183 ligand 7α,25-OHC. Splenic LTi-like ILC3s showed a typical bell-shaped chemotactic response to 7α,25-OHC (Figure 1D), demonstrating that GPR183 is functional in ILC3s. Consistent with high *Gpr183* expression (Figure S2F), splenic CD4^+^ LTi-like ILC3s showed a greater migratory response than other cells to 7α,25-OHC (Figure 1E). Colonic ILC3s and ILC2s also migrated toward 7α,25-OHC *in vitro* (Figure 1F). To confirm that 7α,25-OHC drives ILC3 migration through GPR183, we examined the chemotaxis of *Gpr183*-deficient ILCs. For this purpose, we generated *Gpr183*^−/−^ mice. LTi-like ILC3s lacking *Gpr183* failed to migrate toward 7α,25-OHC (Figure 1D), indicating that ILC3 chemotaxis to oxysterol is GPR183 dependent. We concluded that high GPR183 expression enabled LTi-like ILC3s to migrate toward the chemoattractant oxysterol 7α,25-OHC.

### GPR183 Expression by ILC3s Is Required for the Formation of Colonic Lymphoid Tissues

**Figure 2:**
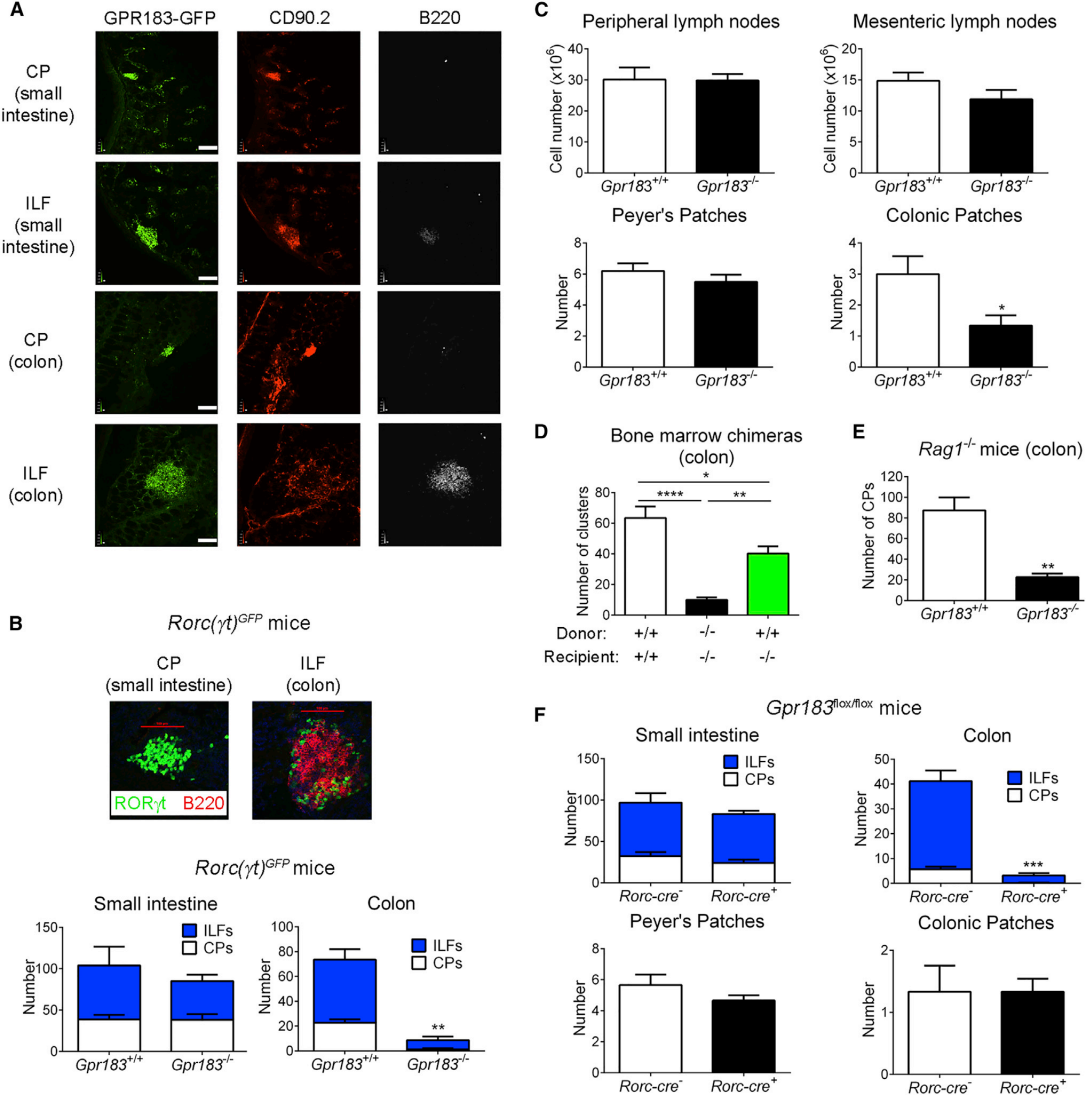
ILC3-Expressed GPR183 Is Required for the Formation of Colonic Lymphoid Tissues (A) Distribution of GFP^+^ cells in the small intestine and colon of *Gpr183*^*GFP*/*+*^ mice. Tissue sections were co-stained with α-CD90.2 and α-B220 Abs. Scale bars (white) represent 100 μm. (B) Number of CPs and ILFs in the small intestine and colon of *Rorc(γt)*^*GFP*^*Gpr183*^+/+^ and *Rorc(γt)*^*GFP*^*Gpr183*^−/−^ mice (n = 3–5). The upper panel shows representative images of a CP and an ILF from *Rorc(γt)*^*GFP*^*Gpr183*^+/+^ mice. Scale bars (red) represent 100 μm. (C) Number of peripheral and mesenteric lymph node cells (n = 6–8), Peyer’s patches (n = 10), and colonic patches (n = 3) from *Gpr183*^+/+^ and *Gpr183*^−/−^ mice. (D) Number of RORγt^+^ clusters in the colon of bone marrow chimeras (n = 5–9). Bone marrow cells from *Gpr183*^+/+^ or *Gpr183*^−/−^ mice were injected into either *Gpr183*^+/+^ or *Gpr183*^−/−^ irradiated recipient mice. (E) Number of CPs in the colon of *Rag1*-deficient *Gpr183*^+/+^ and *Gpr183*^−/−^ mice (n = 5). (F) Number of CPs, ILFs, Peyer’s patches, and colonic patches in *Rorc-cre Gpr183*^flox/flox^ mice and *Gpr183*^flox/flox^ or *Gpr183*^flox/+^ controls (n = 3–6). Data are represented as means ± SEM. ^∗^p < 0.05, ^∗∗^p < 0.01, ^∗∗∗^p < 0.001, ^∗∗∗∗^p < 0.0001 by Student’s t test or one-way ANOVA with Tukey’s post-test (D). Data are representative of or combined from two (F) or three (A–E) experiments. See also Figures S3 and S4.

GPR183 expression in LTi-like ILC3s and B cells, which are known to localize to CPs and ILFs, suggested that GPR183^+^ cells were not randomly distributed in the intestine. To analyze the distribution of GPR183-expressing cells, we prepared gut tissue sections from *Gpr183*^*GFP*/*+*^ mice. We found that GPR183^+^ cells clustered in both CPs (mainly composed of CD90.2^+^ ILCs) and ILFs (also containing B220^+^ B cells) in the colon and small intestine (Figure 2A). The fact that ILC3s with LTi function highly expressed GPR183 led us to hypothesize that GPR183 is required for the development of intestinal lymphoid structures. To explore this hypothesis, we crossed *Gpr183*^−/−^ mice with *Rorc(γt)*^*GFP*^ transgenic mice to visualize and quantify SILTs in frozen sections. Consistent with our hypothesis, the number of CPs and ILFs was markedly lower in the colon of mice lacking *Gpr183* than in co-housed *Gpr183*^+/+^ littermates (Figure 2B). Moreover, the number of colonic patches was also significantly lower in *Gpr183*-deficient mice (Figure 2C). In contrast, CPs and ILFs were present in the small intestine of *Gpr183*^−/−^ mice (Figure 2B). GPR183 was also dispensable for the development of peripheral and mesenteric lymph nodes, as well as Peyer’s patches (Figure 2C).

To confirm that GPR183 expression in hematopoietic cells was required for CP and ILF formation, we generated bone marrow chimeras. We found that the transfer of *Gpr183*^+/+^ bone marrow into *Gpr183*-deficient recipients partially rescued SILT development in the colon (Figure 2D). A full rescue was not expected because LTi-like ILC3s, in contrast to B cells, are partially radioresistant, which created a mixed ILC3 compartment, consisting of donor-derived *Gpr183*^+/+^ and radioresistant *Gpr183*-deficient ILC3s of host origin (Figure S3). The reduction of both CPs and ILFs in *Gpr183*^−/−^ mice suggested that GPR183 expression on ILC3s, rather than on B cells, was required for colonic lymphoid tissue development. To exclude a role of B-cell-expressed GPR183, we bred *Gpr183*^−/−^ mice onto a *Rag1*-deficient background. The number of colonic CPs was significantly lower in *Rag1*- and *Gpr183*-double deficient mice than in *Rag1*-deficient *Gpr183*-sufficient mice (Figure 2E).

Our finding that ILC2s expressed *Gpr183* mRNA (Figure 1B) and migrated toward 7α,25-OHC (Figure 1F) allowed us to predict that ILC2s also reside in colonic lymphoid structures. We confirmed this prediction by staining with α-GATA3 (Figure S4A) and α-KLRG1 antibodies (Abs) (Figure S4B). To determine whether ILC3-expressed GPR183 was required for CP and ILF formation, we generated *Rorc-cre Gpr183*^flox/flox^ mice, where *Gpr183* expression was ablated in ILC3. In these mice, T cells also lacked *Gpr183*, but T cells are known to be dispensable for lymphoid tissue formation in the intestine (Kiss et al., 2011, Tsuji et al., 2008). We found that CP and ILF development in the colon, but not in the small intestine, was severely impaired in *Rorc-cre Gpr183*^flox/flox^ mice, whereas Peyer’s and colonic patches were not affected (Figure 2F). Collectively, our data demonstrate that GPR183-expressing cells reside in gut lymphoid structures and that GPR183 expression by ILC3s is crucial for the formation of CPs and ILFs in the colon.

### GPR183 and 7α,25-OHC Promote ILC Localization to CPs

**Figure 3:**
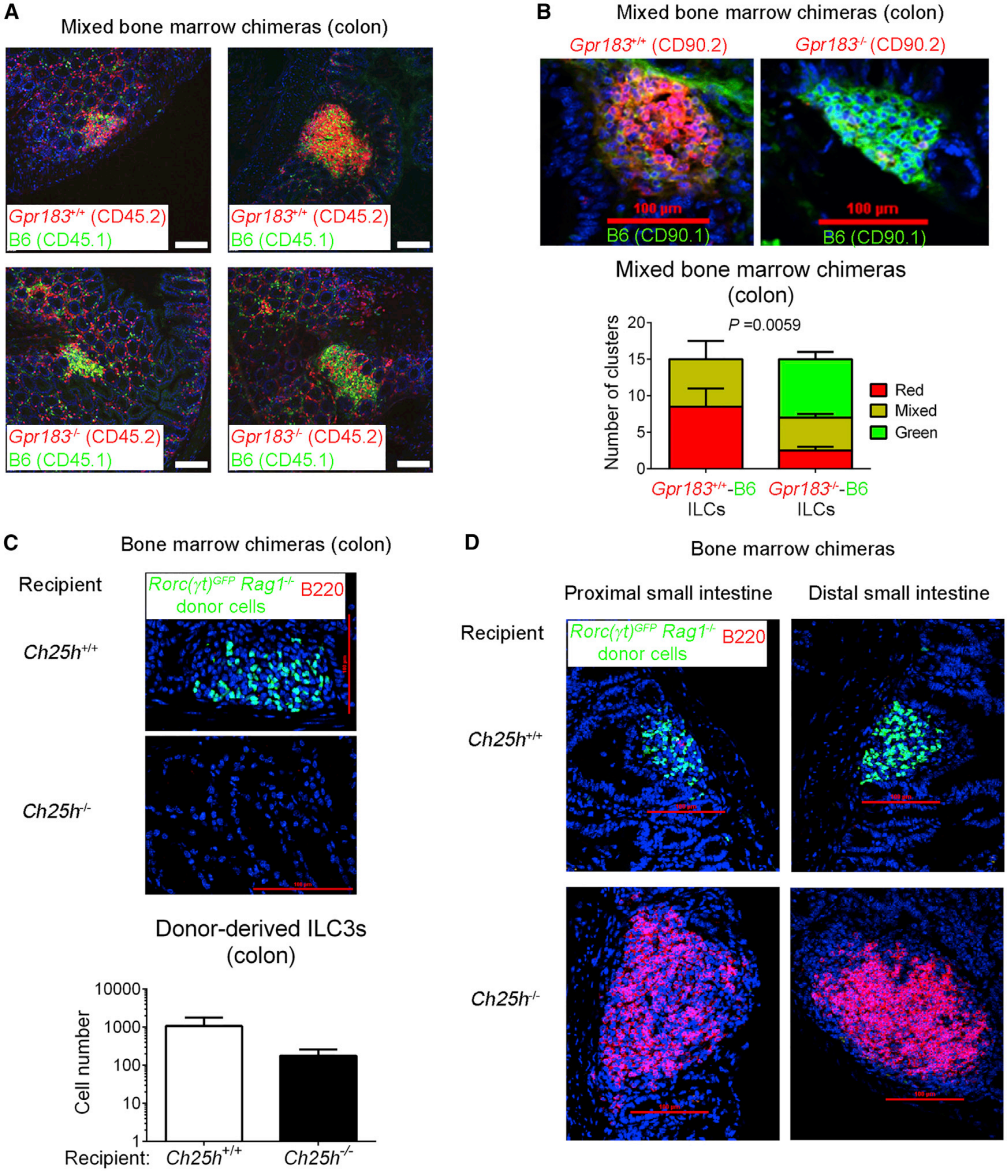
GPR183 and 7α,25-OHC Promote ILC3 Localization to CPs and ILFs (A) Distribution of *Gpr183*^+/+^ and *Gpr183*-deficient hematopoietic cells in the colon of mixed bone marrow chimeras. Bone marrow cells from *Gpr183*^+/+^ or *Gpr183*^−/−^ mice (CD45.2^+^) were mixed 9:1 with B6 cells (CD45.1^+^) and injected into irradiated *Rag1*^−/−^ recipients (CD45.1^+^) for the generation of bone marrow chimeras. Sections were stained for detection of *Gpr183*^+/+^ and *Gpr183*^−/−^ cells (CD45.2, red) or B6 cells (CD45.1, green). Nuclei were visualized by DAPI staining (blue). Scale bars on the right (white) represent 100 μm. (B) Distribution of *Gpr183*^+/+^ and *Gpr183*-deficient ILCs in the colon of mixed bone marrow chimeras. Bone marrow cells from *Gpr183*^+/+^ or *Gpr183*^−/−^ mice (CD90.2^+^) were mixed 9:1 with B6 cells (CD90.1^+^) and injected into irradiated B6 recipients (CD90.1^+^) for the generation of bone marrow chimeras. Colon sections were stained for detection of *Gpr183*^+/+^ and *Gpr183*^−/−^ (CD90.2, red) or B6 (CD90.1, green) ILCs. Scale bars (red) represent 100 μm. The lower panel shows the number of clusters in *Gpr183*^+/+^-B6 and *Gpr183*^−/−^-B6 chimeras consisting of CD90.2^+^ (red) or CD90.1^+^ (green) ILCs. Data are represented as means ± SEM. p value by two-way ANOVA. (C and D) Distribution of donor-derived ILC3s (RORγt-GFP^+^, green) in the colon (C) and small intestine (D) of bone marrow chimeras. Bone marrow cells from *Rag1*-deficient *Rorc(γt)*^*GFP*^ transgenic mice were injected into irradiated *Ch25h*^+/+^ or *Ch25h*^−/−^ recipients for the generation of bone marrow chimeras. Sections were co-stained for detection of B cells (B220^+^, red). Scale bars (red) represent 100 μm. The lower panel in (C) shows the number of donor-derived ILC3s in the colon of *Ch25h*^+/+^ or *Ch25h*^−/−^ hosts. Data are representative of or combined from two (B–D) or three (A) experiments. See also Figure S5.

Next, we wanted to establish how GPR183 regulates CP and ILF formation in the colon. On the basis of the fact that GPR183 controls immune cell migration, we asked whether GPR183 is required for ILC3 recruitment to CPs. To address this question, we generated mixed bone marrow chimeras by sub-lethally irradiating *Rag1*-deficient mice (CD45.1^+^) and reconstituting them with a 9:1 mixture of *Gpr183*^+/+^ or *Gpr183*^−/−^ (CD45.2^+^) and C57BL/6 (B6) (CD45.1^+^) bone marrow cells (Figure S5A). This experimental setup allowed us to study *Gpr183*-deficient and -sufficient cells in the same environment, thereby excluding any cell-extrinsic effects on cell distribution. As expected, CPs and ILFs in mixed *Gpr183*^+/+^-B6 chimeras consisted mainly of CD45.2^+^ *Gpr183*^+/+^ cells (Figure 3A). In contrast, *Gpr183*-deficient hematopoietic cells failed to localize to CPs and ILFs in mixed *Gpr183*^−/−^-B6 chimeras, and accordingly, CPs and ILFs in these chimeras mostly contained CD45.1^+^ B6 cells (Figure 3A). To more specifically address whether ILC3 migration to CPs and ILFs is dependent on GPR183, we employed a similar mixed bone marrow chimera approach, by using the congenic marker CD90 (Thy1) instead of CD45 (Figure S5B), given that CD90 labels ILCs more specifically than CD45. Notably, donor-derived *Gpr183*^−/−^ ILCs (CD90.2^+^) were largely excluded from CPs, whereas ILCs derived from *Gpr183*^+/+^ bone marrow (CD90.2^+^) selectively localized to CPs (Figure 3B). These observations demonstrate that GPR183 acts in a cell-autonomous manner to promote ILC localization to colonic CPs and ILFs.

To determine whether ILC positioning to CPs and ILFs is directed by 7α,25-OHC, we assessed the spatial distribution of ILC3s in mice lacking *Ch25h*. For this purpose, we reconstituted either *Ch25h*^+/+^ or *Ch25h*^−/−^ recipients with bone marrow from *Rag1*-deficient *Rorc(γt)*^*GFP*^ transgenic mice (Figure S5C). Immunofluorescence microscopy showed that donor-derived GFP^+^ ILC3s localized to colonic CPs in *Ch25h*^+/+^ mice, whereas donor-derived ILC3s were unable to do so in *Ch25h*-deficient hosts (Figure 3C). In the absence of *Ch25h*, donor-derived ILC3s also failed to migrate to CPs in the small intestine (Figure 3D). In contrast to in the colon, however (Figure 3C), host B cells in the small intestine formed enlarged lymphoid clusters, despite the ILC3 localization defect (Figure 3D). These findings suggest that lymphoid-tissue-inducing activity of B cells compensates for impaired ILC3 recruitment when GPR183 ligand is lacking, which subsequently allows lymphoid tissue formation in the small intestine. Combined, these data demonstrate that GPR183 and its ligand promote colonic SILT development by directing LTi-like ILC3s to sites of CP formation.

### GPR183 Enhances IL-22 Production by Colonic ILC3s

Next, we asked whether altered ILC3 positioning in the absence of GPR183 causes defects in the development, homeostasis, or function of ILC3s. There was no general developmental defect, given that all ILC subsets were present in the colon of *Gpr183*^−/−^ mice (Figures S6A and S6B). Furthermore, flow-cytometric analysis revealed that the number of individual ILC3 subsets was not significantly different between *Gpr183*^+/+^ mice and mice lacking *Gpr183* (Figure S6C). We next investigated the expression of lymphotoxin, the key factor for lymphoid organogenesis. To exclude a lymphocyte source of lymphotoxin, we performed this analysis in *Rag1*-deficient *Gpr183*^−/−^ mice. Lymphotoxin beta (*Ltb*) mRNA expression in the colon was ∼3-fold lower in *Rag1*^−/−^ mice lacking *Gpr183* than in *Gpr183*^+/+^ *Rag1*^−/−^ mice (Figure S6D). CP and ILF formation is also dependent on aryl hydrocarbon receptor (AHR) (Kiss et al., 2011, Lee et al., 2011). However, the amount of *Ahr* mRNA in the colon was not different between *Rag1*-deficient *Gpr183*^+/+^ and *Gpr183*^−/−^ mice (Figure S6D). In contrast to whole colon tissue, purified *Gpr183*-deficient ILC3s from *Rag1*^−/−^ mice expressed normal amounts of *Lta* and *Ltb* mRNA (Figure S6E). The membrane-bound form of lymphotoxin (LTα_1_β_2_) is required for lymphoid tissue formation, and we therefore examined LTα_1_β_2_ surface expression by using a LTβ receptor (LTβR)-Fc fusion protein. This experiment was performed with ILCs from mesenteric lymph nodes given that we could not detect surface LTα_1_β_2_ in *Gpr183*^+/+^ ILC3s from the colon, most likely because the cell-isolation procedure led to a loss of LTα_1_β_2_ from the cell surface. *Gpr183*-deficient CD4^+^ LTi-like ILC3s had significantly lower amounts of LTα_1_β_2_ on the cell surface than their *Gpr183*-sufficient counterparts (Figure S6F). Together, our data suggest that *Gpr183* deficiency causes impaired colonic lymphoid tissue formation mainly because of the inability of LTi-like ILC3s to migrate to CPs and ILFs rather than an intrinsic defect in lymphotoxin expression by ILC3s.

ILFs are important sites of immunoglobulin A (IgA) production (Tsuji et al., 2008), and the reduction of colonic lymphoid structures indicates that IgA production might be impaired in mice lacking *Gpr183*. To explore this possibility, we measured IgA in either untreated or T-cell-depleted *Gpr183*^+/+^ and *Gpr183*^−/−^ mice. IgA concentrations in serum and feces were not significantly different between *Gpr183*-deficient and *Gpr183*-sufficient mice (Figure S6G), most likely as a result of compensatory IgA production by small intestinal lymphoid structures, which were not affected by *Gpr183* deficiency (Figures 2B and 2C).

**Figure 4:**
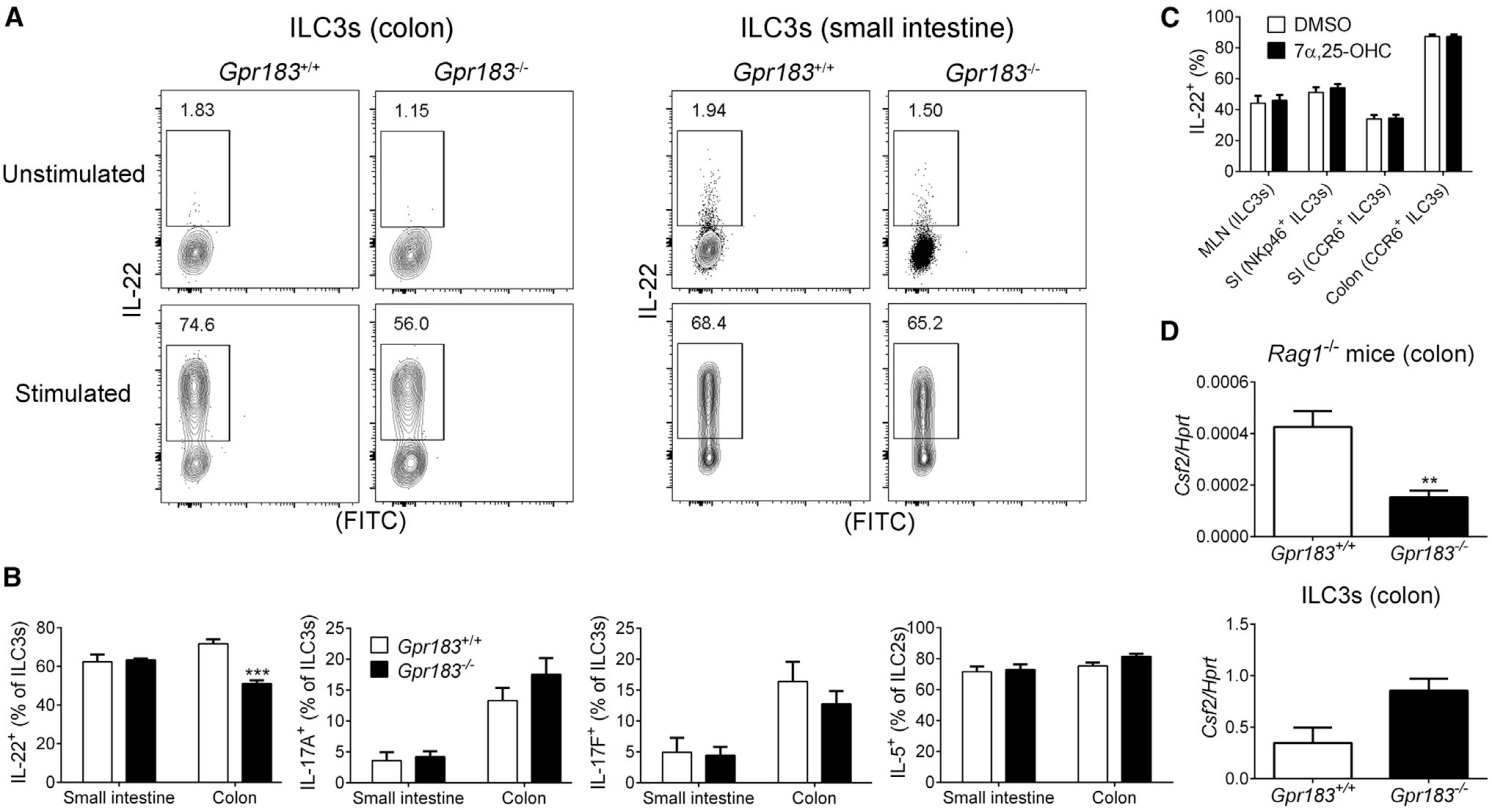
GPR183 Enhances IL-22 Production by Colonic ILC3s (A) IL-22 production by ILC3s from the colon or small intestine of *Gpr183*^+/+^ and *Gpr183*^−/−^ mice. Numbers indicate cell frequencies. (B) Frequency of intestinal ILC3s producing IL-17A, IL-17F, and IL-22 and frequency of ILC2s producing IL-5 from *Gpr183*^+/+^ and *Gpr183*^−/−^ mice (n = 3–4). (C) Frequency of IL-22-producing ILC3s from mesenteric lymph nodes (MLN), small intestine (SI), and colon of B6 mice after stimulation with IL-1β and IL-23 in the presence of solvent control dimethyl sulfoxide (DMSO) or 10 nM 7α,25-OHC (n = 10). (D) *Csf2* mRNA expression in the colon (top; n = 8–9) or in sorted colonic ILC3s (bottom; n = 2) from *Gpr183*^+/+^ and *Gpr183*^−/−^ mice on a *Rag1*-deficient background. mRNA expression was normalized to *Hprt*. Data are represented as means ± SEM. ^∗∗^p < 0.01, ^∗∗∗^p < 0.001 by Student’s t test. Data are representative of or combined from two experiments. See also Figure S6.

We next assessed whether *Gpr183* deficiency alters the ability of ILCs to produce cytokines. IL-22 production by *Gpr183*-deficient ILC3s from the colon was significantly lower than production by their *Gpr183*^+/+^ counterparts (Figures 4A and 4B). In contrast, IL-22 production by *Gpr183*-deficient ILC3s from the small intestine was unaffected (Figures 4A and 4B). Most likely, impaired IL-22 production by *Gpr183*^−/−^ ILC3s from the colon was secondary to the ILC3 migration defect and reduction of colonic CPs and ILFs when *Gpr183* was lacking. Accordingly, 7α,25-OHC failed to directly modulate IL-22 production by ILC3s *in vitro* (Figure 4C). Moreover, the lack of *Gpr183* had no effect on IL-5 and IL-17 production by intestinal ILC2s and ILC3s, respectively (Figure 4B). Finally, *Gpr183*^−/−^ mice (on a *Rag1*^−/−^ background) expressed less colony stimulating factor 2 (*Csf2*) mRNA in the colon than their *Gpr183*^+/+^ counterparts, whereas purified *Gpr183*-deficient ILC3s had slightly higher *Csf2* expression than *Gpr183*^+/+^ ILC3s (Figure 4D). In summary, these data show that GPR183 is dispensable for IgA production but promotes IL-22 production by colonic ILC3s.

### Fibroblastic Stromal Cells Provide a Local Source of 7α,25-OHC

**Figure 5:**
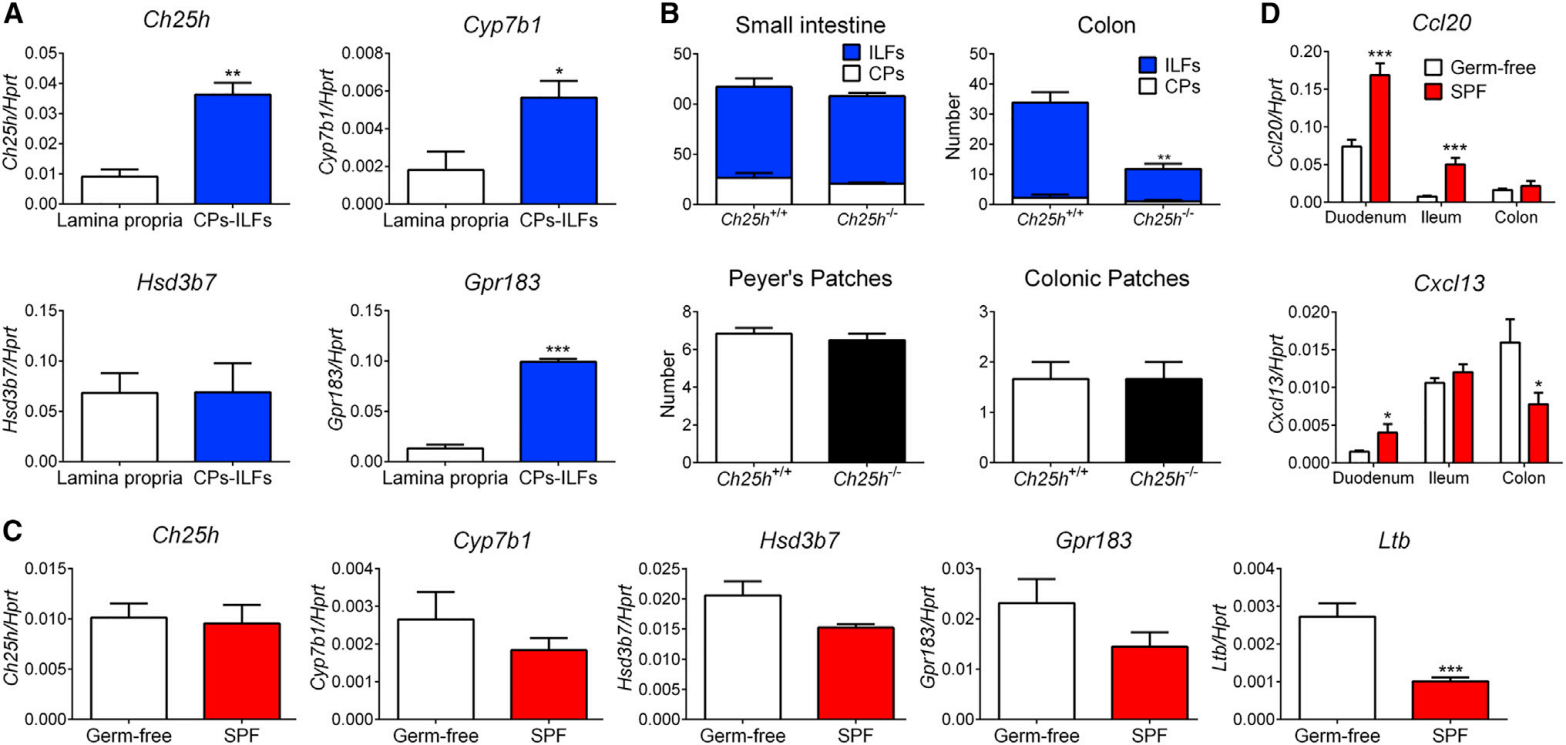
Microbiota-Independent Local 7α,25-OHC Production Is Necessary for Colonic CP and ILF Formation (A) *Ch25h*, *Cyp7b1*, *Hsd3b7*, and *Gpr183* mRNA expression in the colon of human *CD2*^*GFP*^ transgenic mice (n = 3). mRNA expression was compared in micro-dissected CPs and ILFs (CPs-ILFs) versus micro-dissected lamina propria. (B) Number of CPs, ILFs, Peyer’s patches, and colonic patches in *Ch25h*^+/+^ and *Ch25h*^−/−^ mice (n = 3–6). (C) *Ch25h*, *Cyp7b1*, *Hsd3b7*, *Gpr183*, and *Ltb* mRNA expression in the colon of germ-free and specific-pathogen-free (SPF) mice (n = 6). (D) *Ccl20* and *Cxcl13* mRNA expression in the intestine of germ-free and SPF mice (n = 6). Data are represented as means ± SEM. ^∗^p < 0.05, ^∗∗^p < 0.01, ^∗∗∗^p < 0.001 by Student’s t test. Data are representative of or combined from two experiments. See also Figure S7.

GPR183 and its ligand 7α,25-OHC directed ILC3 migration to CPs and ILFs (Figure 3). This allowed us to predict that production of 7α,25-OHC should take place in CPs and ILFs and thereby attract GPR183^+^ cells. To test this prediction, we measured 7α,25-OHC-synthesizing enzymes in micro-dissected colonic CPs and ILFs (Figure S7A). Consistent with our hypothesis, we found that mRNA for the GPR183-ligand-synthesizing enzymes CH25H and CYP7B1 was significantly enriched in CPs and ILFs, whereas expression of the ligand-degrading enzyme HSD3B7 was not different between the lamina propria and CPs and ILFs (Figure 5A). Similar results were obtained for colonic (Figure S7B) and Peyer’s (Figure S7C) patches. These data suggest that local 7α,25-OHC production takes place in colonic lymphoid structures, and we therefore asked whether 7α,25-OHC promotes CP and ILF formation. To investigate this possibility, we used mice lacking *Ch25h* and therefore 7α,25-OHC. The number of colonic CPs and ILFs was significantly lower in *Ch25h*-deficient than in *Ch25h*^+/+^ mice (Figure 5B), demonstrating that 7α,25-OHC is essential for lymphoid organogenesis in the colon. In contrast, the formation of lymphoid structures in the small intestine and colonic patches was not affected by the absence of *Ch25h* (Figure 5B). Overall, colonic lymphoid tissue formation was less affected in *Ch25h*- than in *Gpr183*-deficient mice, most likely as a result of compensation by the alternative GPR183 ligand 7α,27-OHC, which is generated independently of CH25H (Lu et al., 2017).

Next, we asked which signals from the microenvironment regulate 7α,25-OHC production. Among environmental factors, the microbiota plays a pivotal role in intestinal homeostasis and immunity. Using germ-free mice, we found that *Gpr183*, *Ch25h*, and *Cyp7b1* mRNA expression in the colon was independent of the microbiota (Figure 5C). Furthermore, *Ltb* mRNA expression in the colon was repressed by the microbiota, consistent with the observation that, in contrast to ILF formation in the small intestine, ILF formation in the colon is inhibited by the microbiota (Buettner and Lochner, 2016, Randall and Mebius, 2014). Accordingly, the microbiota promoted expression of C-C motif chemokine ligand 20 (*Ccl20*) and C-X-C motif chemokine ligand 13 (*Cxcl13*) mRNA only in the small intestine, but not in the colon (Figure 5D). Together, these data suggest that microbiota-independent local generation of 7α,25-OHC is necessary for colonic CP and ILF formation.

**Figure 6:**
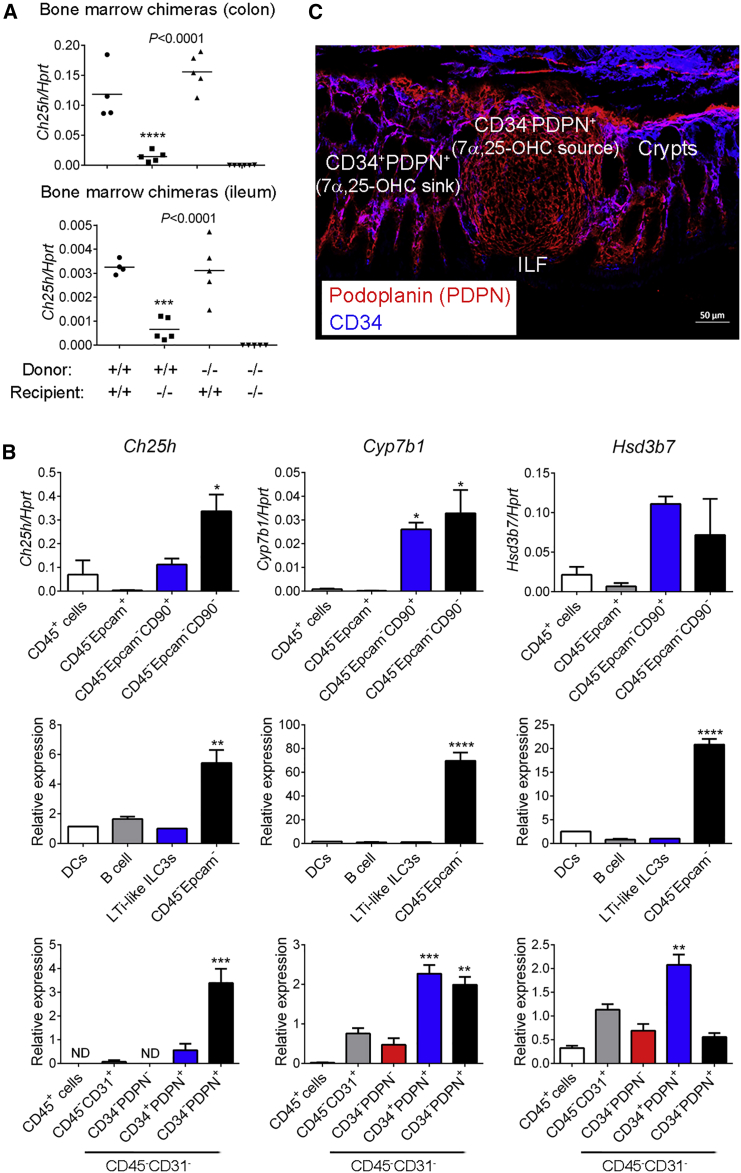
Fibroblastic Stromal Cells Provide a Source of 7α,25-OHC (A) *Ch25h* mRNA expression in the colon and ileum of bone marrow chimeras (n = 4–6). Chimeras were generated with *Ch25h*^+/+^ and *Ch25h*^−/−^ mice as bone marrow donors and recipients as indicated. (B) *Ch25h*, *Cyp7b1*, and *Hsd3b7* mRNA expression in the indicated purified cell populations from the colon of B6 mice (n = 1–7). (C) Immunofluorescence microscopy of colon sections from B6 mice were stained with Abs against podoplanin (PDPN) (red) and CD34 (blue). Scale bar represents 50 μm. Data are represented as means ± SEM. ^∗^p < 0.05, ^∗∗^p < 0.01, ^∗∗∗^p < 0.001, ^∗∗∗∗^p < 0.0001 by one-way ANOVA with Tukey’s post-test. Data are representative of or combined from two or three experiments. See also Figure S7.

Finally, we aimed to identify the cellular source of the GPR183 ligand 7α,25-OHC in the colon. To distinguish between hematopoietic- and non-hematopoietic-expressed CH25H, we generated bone marrow chimeras. *Ch25h*^+/+^ recipients injected with *Ch25h*^−/−^ bone marrow had colonic *Ch25h* mRNA expression similar to that of *Ch25h*^+/+^ mice transplanted with *Ch25h*^+/+^ bone marrow (Figure 6A). However, *Ch25h* mRNA expression in *Ch25h*^−/−^ mice was not rescued by the transfer of *Ch25h*^+/+^ bone marrow (Figure 6A). Similar results were obtained for the small intestine (Figure 6A). These findings demonstrate that hematopoietic-derived CH25H does not contribute to CH25H expression and that, instead, radio-resistant cells provide the main source of CH25H in the intestine. Fibroblastic stromal cells that are specialized in interacting with immune cells are found in lymphoid structures, and we hypothesized that radio-resistant stromal cells are a likely source of the GPR183 ligand 7α,25-OHC in CPs and ILFs. Consistent with this hypothesis, *Ch25h* and *Cyp7b1* mRNA expression was much higher in non-hematopoietic-non-epithelial cells (CD45^−^Epcam^−^) than in LTi-like ILC3s, DCs, B cells, and epithelial cells (CD45^−^Epcam^+^) isolated from the colon (Figure 6B). Next, we measured expression of oxysterol-generating enzymes in different populations of colonic CD45^−^ cells as previously described (Stzepourginski et al., 2017). These experiments revealed that CD34^−^ podoplanin (PDPN, also known as gp38)^+^ stromal cells abundantly expressed the 7α,25-OHC-synthesizing enzymes CH25H and CYP7B1 (Figure 6B) and localized to ILFs (Figure 6C). In contrast, CD34^+^PDPN^+^ stromal cells, which were located outside of ILFs (Figure 6C), had higher expression of the GPR183-ligand-degrading enzyme HSD3B7 (Figure 6B). Together, these results suggest that ILF-resident CD34^−^PDPN^+^ fibroblastic stromal cells locally produce 7α,25-OHC and that neighboring CD34^+^PDPN^+^ stromal cells act as a sink for oxysterols and thereby create a 7α,25-OHC gradient between ILFs and the surrounding lamina propria. Finally, using *Cxcl13*-EYFP reporter mice (Onder et al., 2017), we found that CXCL13^+^PDPN^+^CD31^−^ stromal cells clustered in ILFs (Figures S7D–S7F) and expressed high amounts of *Ch25h* and *Cyp7b1* mRNA (Figure S7G). These data reinforce our concept that the GPR183 ligand 7α,25-OHC is produced by fibroblastic stromal cells that reside in CPs and ILFs. We conclude that production of 7α,25-OHC by fibroblastic stromal cells provides a critical local signal for the development of lymphoid structures in the colon.

### GPR183 Expression in Innate Immune Cells Promotes Colitis

**Figure 7:**
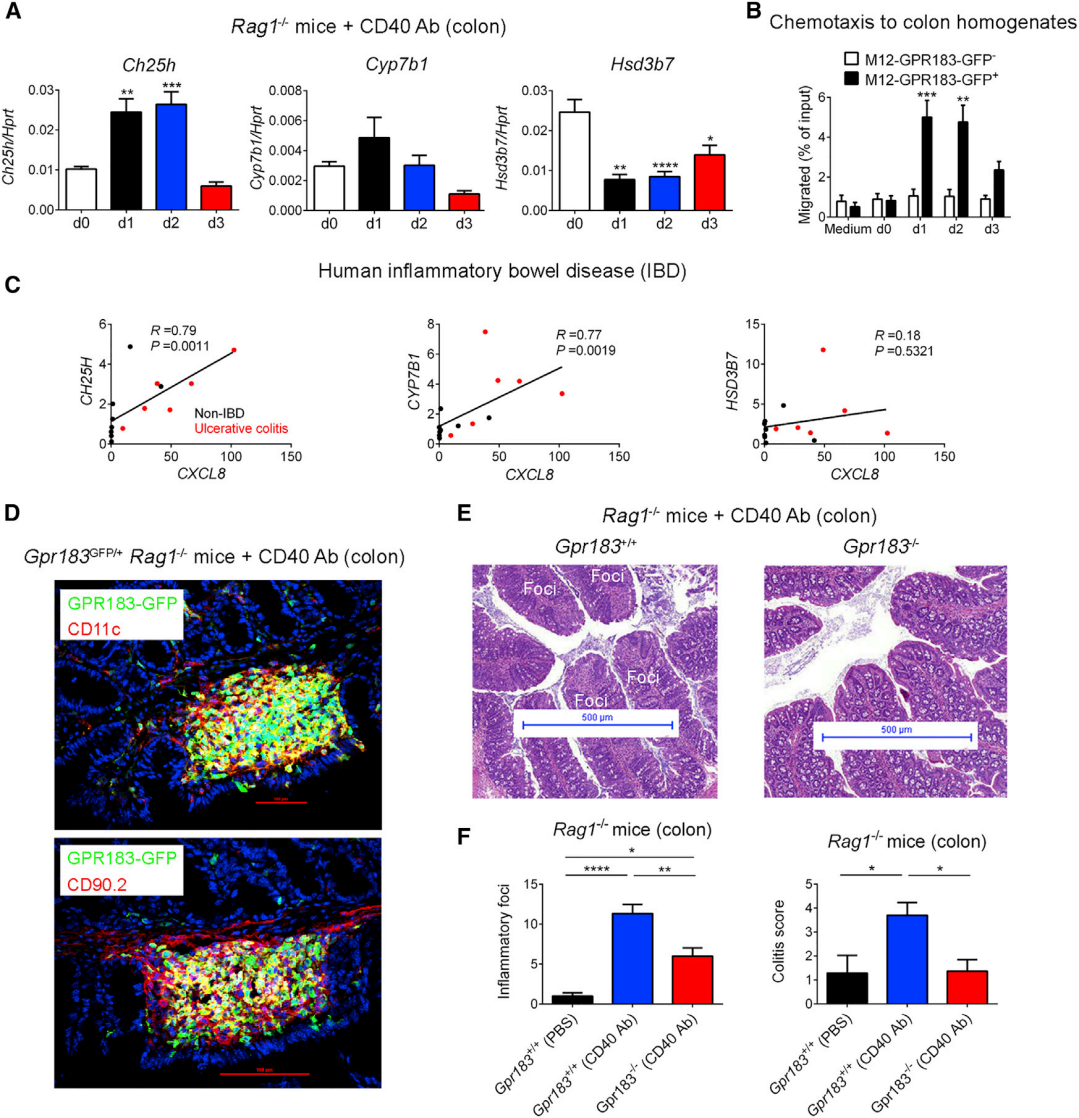
GPR183 Expressed on Innate Immune Cells Promotes Colitis (A) Colonic *Ch25h*, *Cyp7b1*, and *Hsd3b7* mRNA expression in *Rag1*^−/−^ mice injected with 100 μg CD40 Ab (n = 6–7). d, day. (B) Transwell migration of GPR183-transduced B cell line (M12-GPR183-GFP) to colon homogenates from CD40-Ab-treated *Rag1*^−/−^ mice (n = 4). Chemotaxis of GFP^−^ M12 cells was used as a negative control. (C) Correlation of *CH25H*, *CYP7B1*, and *HSD3B7* with *CXCL8* mRNA expression in the colon of healthy controls (black dots; n = 8) and patients with ulcerative colitis (red dots; n = 6). (D) Immunofluorescence microscopy of proximal colon from *Rag1*-deficient *Gpr183*^*GFP/+*^ mice 7 days after CD40 Ab injection. Sections were co-stained for detection of nuclei (DAPI) and myeloid cells (CD11c) or ILCs (CD90.2). Inflammatory foci are shown. Scale bars (red) represent 100 μm. (E) H&E staining of proximal colon from *Rag1*-deficient *Gpr183*^+/+^ and *Gpr183*^−/−^ mice 7 days after CD40 Ab treatment. Inflammatory foci at the tip of colonic folds are indicated. Scale bars (blue) represent 500 μm. (F) Number of inflammatory foci and colitis score in CD40-Ab-treated *Gpr183*^+/+^*Rag1*^−/−^ and *Gpr183*^−/−^*Rag1*^−/−^ mice (n = 7–15). PBS-treated *Gpr183*^+/+^*Rag1*^−/−^ mice were used as controls. Data are represented as means ± SEM. ^∗^p < 0.05, ^∗∗^p < 0.01, ^∗∗∗^p < 0.001, ^∗∗∗∗^p < 0.0001 by one-way ANOVA with Tukey’s post-test. Data are representative of or combined from two experiments.

Our results so far have established a role for the oxysterol-GPR183 pathway in lymphoid tissue formation in the steady-state colon. Consequently, we asked whether this pathway has a similar function during inflammation, which causes immune cell infiltration and tissue remodeling. This seemed particularly relevant given that hyperplastic lymphoid aggregates occur in human inflammatory bowel disease (IBD) (Buettner and Lochner, 2016), and polymorphisms in *Gpr183* have been associated with IBD (Jostins et al., 2012). We first examined whether local inflammation affects the production of GPR183 ligands in the colon. For this purpose, we used a model of innate colitis in which intestinal inflammation is induced in T- and B-cell-deficient mice by the administration of an agonistic CD40 Ab (Uhlig et al., 2006). Compared with PBS-treated *Rag1*^−/−^ mice, mice treated with CD40 Ab showed rapidly increased *Ch25h* mRNA expression within 24 hr but repressed *Hsd3b7* expression (Figure 7A). This pattern allowed us to predict that the amount of 7α,25-OHC in the colon would increase in response to CD40-Ab-induced inflammation. To test this prediction, we quantified GPR183 ligand activity in the colon by a bioassay based on the ligand-induced chemotaxis of the GPR183-transduced M12 B cell line (Kelly et al., 2011). GPR183 ligand activity was induced by CD40 Ab treatment, given that the chemotactic response of GPR183-GFP^+^ M12 cells to colon homogenates rapidly increased within 24 hr after CD40 Ab injection (Figure 7B). Our results suggest that colonic inflammation activates the oxysterol-GPR183 pathway through increased production of the GPR183 ligand 7α,25-OHC. Accordingly, there was a significant correlation between *CH25H* and *CYP7B1* expression and colonic inflammation (as measured by *CXCL8* expression) in humans with ulcerative colitis (Figure 7C).

In the CD40-Ab-induced colitis model, mobilization of ILC3s from CPs into adjacent tissue and recruitment of inflammatory monocytes (Pearson et al., 2016) result in the formation of characteristic inflammatory foci. The timing of inflammation-induced production of GPR183 ligands coincided with the movement of ILC3s out of CPs and the formation of inflammatory foci. We therefore used *Rag1*-deficient *Gpr183*^*GFP*/*+*^ mice to examine whether GPR183^+^ cells localize to inflammatory infiltrates. Fluorescence microscopy of inflamed colon tissue showed that CD40-Ab-induced inflammatory foci at day 7 contained GPR183-expressing myeloid cells and ILCs (Figure 7D). These findings raise the possibility that GPR183-dependent immune cell migration promotes intestinal inflammation. To determine whether GPR183-expressing cells contribute to colitis, we treated *Rag1*-deficient *Gpr183*^−/−^ and littermate *Gpr183*^+/+^ mice with CD40 Ab and assessed inflammation by histology on day 7. As expected, *Gpr183*^+/+^*Rag1*^−/−^ mice treated with CD40 Ab showed numerous characteristic inflammatory foci in the proximal colon (Figure 7E). In contrast, *Rag1*^−/−^ mice lacking *Gpr183* had significantly fewer CD40-induced inflammatory infiltrates than *Gpr183*-sufficient controls (Figures 7E and 7F). Furthermore, CD40-treated *Gpr183*^−/−^*Rag1*^−/−^ mice developed only mild colitis with an inflammation score similar to that of PBS-treated *Gpr183*^+/+^*Rag1*^−/−^ mice (Figure 7F). Overall, our data demonstrate that inflammatory signals stimulate local oxysterol production and cause colitis through GPR183-dependent activation of ILC migration, myeloid cell recruitment, and tissue remodeling.

## Discussion

Little is known regarding how ILCs directly sense signals from their environment and which signaling pathways enable ILCs to perform tissue remodeling. Our work demonstrates that LTi-like ILC3s were controlled by oxysterols that signal through the receptor GPR183. This instructs ILC3 positioning and lymphoid tissue formation in the colon. We propose a model where local generation of 7α,25-OHC by fibroblastic stromal cells attracts GPR183-expressing LTi-like ILC3s to sites of CP formation. This process positions LTα_1_β_2_^+^ ILC3s for crosstalk with LTβR^+^ stromal cells, which promotes the recruitment of GPR183-expressing B cells, to complete ILF formation. Moreover, our study provides information on the mechanisms that control the spatial and functional compartmentalization of ILC3s in the intestine. After homing to the intestine, ILC3s segregate into two distinct locations. Localization of NKp46^+^ ILC3s to the villi of the lamina propria occurs in a CXCL16-CXCR6-dependent manner, which supports epithelial defense through the production of IL-22 (Satoh-Takayama et al., 2014). We have shown that GPR183 and its ligand 7α,25-OHC position LTi-like ILC3s to colonic CPs, where these cells promote lymphoid tissue formation.

Previous studies have established an essential function for GPR183 and its ligand 7α,25-OHC in lymphoid organs and humoral immunity (Gatto et al., 2009, Gatto et al., 2013, Hannedouche et al., 2011, Li et al., 2016, Liu et al., 2011, Pereira et al., 2009, Yi and Cyster, 2013, Yi et al., 2012). We have now demonstrated a role for GPR183 and oxysterols in lymphoid tissue development in the large intestine. Thus, our study has established a broader function of the oxysterol-GPR183-ligand-receptor system by linking GPR183-mediated cell positioning to tissue reorganization during steady-state homeostasis and inflammation. Whereas the signals regulating the formation of SILTs in the small intestine are well known, those in the colon have remained poorly understood. As in the small intestine, LTi-like ILC3s and LTα_1_β_2_ are required for colonic lymphoid organogenesis. However, the microbiota and receptor activator of NF-κB ligand (RANKL)-RANK, CXCL13-CXCR5, and CCL20-CCR6 are required for ILF development only in the small intestine (Buettner and Lochner, 2016, Randall and Mebius, 2014). Accordingly, we have described a colon-specific pathway governing the postnatal development of lymphoid tissues.

One question relates to our finding that oxysterol-GPR183 signaling is critical for CP and ILF formation in the colon but dispensable in the small intestine. GPR183 and its ligand were expressed in both the small and large intestines. Moreover, 7α,25-OHC was required for ILC3 migration to CPs not only in the colon but also in the small intestine. Despite this, lymphoid tissue development in the small intestine was normal in mice lacking 7α,25-OHC. Together, our observations indicate that, as a compensatory response, B cells form lymphoid follicles when ILC3s are unable to migrate to CPs as a result of a lack of GPR183 ligand. This notion is supported by previous studies demonstrating lymphoid-tissue-inducing activity of B cells and compensatory lymphoid tissue formation when ILC3s are absent or when LTβR signaling is blocked *in utero* (Buettner and Lochner, 2016). This process must be driven by factors that operate specifically in the small intestine, because we observed compensatory B cell cluster formation only in the small intestine, but not in the colon. Such factors are the microbiota, CCL20, and CXCL13, which are all dispensable for ILC3 recruitment and CP formation but essential for B cell recruitment and ILF formation in the small intestine (Buettner and Lochner, 2016). In conclusion, we favor the concept that the 7α,25-OHC-GPR183 pathway is active, but not absolutely required for lymphoid tissue formation, in the small intestine because other redundant factors are sufficient in the absence of GPR183 ligand.

Recent studies support the concept that dietary metabolites, such as retinoids and AHR ligands, regulate ILC3 function. These metabolites act through intracellular nuclear receptors that function as transcription factors, thereby controlling ILC activity through the stimulation of specific transcriptional programs. We have now shown that endogenous metabolites derived from cholesterol control ILC3 function by binding to a cell-surface receptor (GPR183). Previously, it was found that 7α,27-OHC and 7β,27-OHC can act as endogenous RORγt agonists and thereby regulate Th17 differentiation (Soroosh et al., 2014). More recently, it was reported that CYP51-dependent intermediates in cholesterol biosynthesis are ligands for RORγt (Santori et al., 2015). Chemically distinct cholesterol metabolites can therefore be sensed intracellularly through RORγt (modulating cell differentiation) and extracellularly through GPR183 (controlling ILC3 migration).

ILC3s have been implicated in gut inflammation in various mouse models of colitis (Buonocore et al., 2010, Powell et al., 2012, Vonarbourg et al., 2010) as well as in humans with IBD (Geremia et al., 2011). Furthermore, intestinal inflammation is associated with the expansion of lymphoid structures in the gut, and hyperplastic lymphoid aggregates have been observed in human IBD (Buettner and Lochner, 2016). We demonstrated that GPR183 promotes lymphoid tissue formation by ILC3s not only during steady state but also during inflammation. Our findings support the notion that GPR183 stimulates pro-inflammatory ILC3 activity in the colon by triggering an inflammatory migratory response that is reminiscent of the postnatal clustering of ILC3s during the formation of CPs and ILFs. Interestingly, a *Gpr183* polymorphism has been associated with IBD in humans (Jostins et al., 2012), supporting the idea that GPR183 promotes intestinal inflammation.

In summary, our study adds to our understanding of a pathway that regulates ILC3 migration, lymphoid tissue organogenesis, and colitis. GPR183 is a GPCR, a class of molecules that are involved in many diseases while being excellent drug targets. Therefore, our findings not only elucidate a molecular mechanism underlying ILC3 function but also point toward a possible therapeutic target for the treatment of human IBD.

## STAR★Methods

### Key Resources Table

**Table:** 

REAGENT or RESOURCE	SOURCE	IDENTIFIER
**Antibodies**		

Anti-mouse CD3 (145-2C11)-FITC	Biolegend	Cat#100305
Anti-mouse CD3 (145-2C11)-PE-Cy7	Biolegend	Cat#100320
Anti-mouse CD3 (145-2C11)-PerCP-eFluor710	eBioscience	Cat#45-0031-82
Anti-mouse CD3 (17A2)-BV421	Biolegend	Cat#100227
Anti-mouse CD4 (RM4-5)-BUV737	BD Biosciences	Cat#564933
Anti-mouse CD5 (53-7.3)-PE-Cy7	Biolegend	Cat#100622
Anti-mouse CD5 (53-7.3)-PerCP-eFluor710	eBioscience	Cat#45-0051-82
Anti-mouse CD11c (N418)-APC	Biolegend	Cat#117310
Anti-mouse CD11c (N418)-PE-Cy7	Biolegend	Cat#117318
Anti-mouse CD11c (N418)-APC-eFluor780	eBioscience	Cat#47-0114-82
Anti-mouse CD11c (N418)-PB	Biolegend	Cat#117322
Anti-mouse CD31 (390)-PerCP/Cy5.5	Biolegend	Cat#102420
Anti-mouse CD34 (RAM34)-eFluor660	eBioscience	Cat#50-0341-82
Anti-mouse CD45 (30-F11)-FITC	Biolegend	Cat#103108
Anti-mouse CD45 (30-F11)-AF700	Biolegend	Cat#103128
Anti-mouse CD45 (30-F11)-BV650	Biolegend	Cat#103151
Anti-mouse CD45.1 (A20)-AF488	Biolegend	Cat#110718
Anti-mouse CD45.1 (A20)-BUV395	BD Biosciences	Cat#565212
Anti-mouse CD45.2 (104)-FITC	Biolegend	Cat#109806
Anti-mouse CD45.2 (104)-AF647	Biolegend	Cat#109818
Anti-mouse CD90.1 (OX7)-AF488	Biolegend	Cat#202505
Anti-mouse CD90.2 (30-H12)-AF647	Biolegend	Cat#105317
Anti-mouse CD90.2 (30-H12)-APC-Cy7	Biolegend	Cat#105328
Anti-mouse CD90.2 (30-H12)-AF700	Biolegend	Cat#105320
Anti-mouse CD90.2 (30-H12)-PE-Cy7	Biolegend	Cat#105326
Anti-mouse CD90.2 (30-H12)-PerCP	Biolegend	Cat#105322
Anti-mouse CD117 (2B8)-BV421	Biolegend	Cat#105827
Anti-mouse CD127 (A7R34)-FITC	eBioscience	Cat#11-1271-82
Anti-mouse CD127 (A7R34)-PE	Biolegend	Cat#135009
Anti-mouse B220 (RA3-6B2)-FITC	Biolegend	Cat#103206
Anti-mouse B220 (RA3-6B2)-APC	Biolegend	Cat#103212
Anti-mouse B220 (RA3-6B2)-AF647	Biolegend	Cat#103226
Anti-mouse B220 (RA3-6B2)-BV785	Biolegend	Cat#103245
Anti-mouse B220 (RA3-6B2)-PE-Dazzle594	Biolegend	Cat#103258
Anti-mouse B220 (RA3-6B2)-SuperBright600	eBioscience	Cat#63-0452-82
Anti-mouse CCR6 (140706)-PE	R&D Systems	Cat#FAB590P
Anti-mouse CCR6 (140706)-APC	R&D Systems	Cat#FAB590A
Anti-mouse CCR6 (29-2L17)	Biolegend	Cat#129816
Anti-mouse EPCAM (G8.8)-FITC	Biolegend	Cat#118208
Anti-mouse EPCAM (G8.8)-PE	eBioscience	Cat#12-5791-82
Anti-mouse GATA3 (TWAJ)	eBioscience	Cat#14-9966-82
Anti-mouse GATA3 (TWAJ)-PE	eBioscience	Cat#12-9966-42
Anti-mouse IL-5 (TRFK5)-APC	Biolegend	Cat#504305
Anti-mouse IL-17A (EBIO17B7)-PE	eBioscience	Cat#12-7177-81
Anti-mouse IL-17F (9D3.1C8)-AF488	Biolegend	Cat#517005
Anti-mouse IL-22 (IL22JOP)-APC	eBioscience	Cat#17-7222-82
Anti-mouse IL-22 (Poly5164)-AF647	Biolegend	Cat#516406
Anti-mouse KLRG1 (2F1)	BD Biosciences	Cat#562190
Anti-mouse KLRG1 (2F1)-PE	Biolegend	Cat#138408
Anti-mouse KLRG1 (2F1)-BUV395	BD Biosciences	Cat#740279
Anti-mouse MHC class II (M5/114.15.2)-eFluor450	eBioscience	Cat#48-5321-82
Anti-mouse NK1.1 (PK136)-biotin	Biolegend	Cat#108704
Anti-mouse NK1.1 (PK136)-BV711	Biolegend	Cat#108745
Anti-mouse Podoplanin (also known as gp38)	A. Farr (University of Washington, Seattle)	N/A
Anti-mouse Podoplanin (8.1.1)	Biolegend	Cat#127401
Anti-mouse Podoplanin (8.1.1)-PE	Biolegend	Cat#127408
Anti-mouse RORγt (AFKJS-9)	eBioscience	Cat#14-6988-82
Anti-mouse RORγt (B2D)-PE	eBioscience	Cat#12-6981-82
Anti-mouse RORγt (B2D)-PE-eFluor610	eBioscience	Cat#61-6981-82
Anti-mouse RORγt (Q31-378)-PE-CF594	BD Biosciences	Cat#562684
Biotin-SP donkey anti-mouse IgG	Jackson Immunoresearch	Cat#715-065-150
Donkey anti-mouse Fab fragments	Jackson Immunoresearch	Cat#715-007-003
Goat anti-hamster IgG-AF647	ThermoFisher	Cat#A-21451
Goat anti-Syrian hamster IgG-Cy3	Jackson Immunoresearch	Cat#107-165-142
Biotin-SP donkey anti-rat IgG	Jackson Immunoresearch	Cat#712-065-153
Hamster IgG2a isotype control	BD Biosciences	Cat#559339
Rat IgG2a isotype control	BD Biosciences	Cat#553927
Donkey anti-rat IgG-FITC	Jackson Immunoresearch	Cat#712-096-153
AF488-conjugated rabbit anti-fluorescein	ThermoFisher	Cat#A-11090
Donkey anti-rabbit IgG-AF488	ThermoFisher	Cat#A-21206
Rabbit Anti GFP-AF488	Life Technologies	Cat#A21311
Goat anti-mouse IgA	Abcam	Cat#ab97231
Biotinylated goat anti-mouse IgA	Abcam	Cat#ab97233
Mouse IgA	Abcam	Cat#37322
*In vivo* anti-mouse CD40 (FGK45) antibody	Bio X Cell	Cat#BE0016-2
*In vivo* anti-mouse TCRβ (H57-597) antibody	Bio X Cell	Cat#BE0102

**Biological Samples**		

Healthy and IBD human colon tissue	Astrid Lindgren Children’s Hospital, Stockholm	N/A

**Chemicals, Peptides, and Recombinant Proteins**		

7α,25-OHC	Avanti Polar Lipids	Cat#700080P
Collagenase IV	Sigma	Cat#C5138
Collagenase P	Roche	Cat#11215809103
DAPI	Sigma	Cat#10236276001
Dispase I	Roche	Cat#04942078001
DNase I	Sigma	Cat#DN25
DNase I	Applichem	Cat#A3778,0010
Fatty acid-free BSA	Sigma	Cat#A8806
Fixable viability dye-eFluor506	eBioscience	Cat#65-0866
Ionomycin	Sigma	Cat#I9657
Liberase TL	Roche	Cat#05401020001
LTβR-Fc chimera	R&D Systems	Cat#1008-LR-050
Percoll	Sigma	Cat#GE17-0891-01
PMA	Sigma	Cat#P1585
Recombinant IL-1β	eBioscience	Cat#14-8012-62
Recombinant IL-23	eBioscience	Cat#14-8231-63
Recombinant IL-23	R&D Systems	Cat#1887-ML
Streptavidin-PE	Biolegend	Cat#405204
Streptavidin-BV711	Biolegend	Cat#405241
Streptavidin-Cy7	Jackson Immunoresearch	Cat#016-160-084
Trizol	Life Technologies	Cat#15596-018

**Critical Commercial Assays**		

Cell Stimulation Cocktail (plus transport inhibitors)	eBioscience	Cat#00-4975-03
FIX & PERM cell fixation & permeabilization Kit	ThermoFischer	Cat#GAS003
Monensin Protein Transport Inhibitor (GolgiStop)	BD Biosciences	Cat#554724
FoxP3/transcription factor buffer set	eBioscience	Cat#00-5523-00
High-Capacity cDNA reverse transcriptase kit	Applied Biosystems	Cat#4368814
iScript cDNA Synthesis Kit	Biorad	Cat#1778890
RiboPure RNA Purification Kit	Life Technologies	Cat#AM1924
RNeasy Micro Kit	QIAGEN	Cat#74004
SuperScript IV First-Strand Synthesis System	Life Technologies	Cat#18091050

**Experimental Models: Cell Lines**		

Mouse: M12-GPR183-GFP B cell line	Kelly et al., 2011	N/A

**Experimental Models: Organisms/Strains**		

Mouse: C57BL/6 (B6)	National Cancer Institute	N/A
Mouse: B6.SJL-*Ptprc*^*a*^*Pepc*^*b*^/BoyJ (B6-CD45.1)	The Jackson Laboratory	JAX: 002014
Mouse: B6.PL-Thy1.1	The Jackson Laboratory	JAX: 000406
Mouse: Germ-free B6	Karolinska Institutet Core Facility	N/A
Mouse: B6.129S7-*Rag1*^*tm1Mom*^/J (*Rag1*^−/−^)	The Jackson Laboratory	JAX: 002216
Mouse: B6.*Rorc(γt)*^*GFP*^ transgenic	Lochner et al., 2008	N/A
Mouse: B6.*Gpr183*^*GFP/+*^	Pereira et al., 2009	N/A
Mouse: B6.*Gpr183*^−/−^	This paper	N/A
Mouse: B6.*Gpr183*^flox/flox^	This paper	N/A
Mouse: B6.FVB-Tg(*Rorc-cre*)1Litt/J	The Jackson Laboratory	JAX: 022791
Mouse: B6.129S6-*Ch25h*^*tm1Rus*^/J (*Ch25h*^−/−^)	The Jackson Laboratory	JAX: 016263
Mouse: B6.*Cxcl13*-EYFP	Onder et al., 2017	N/A

**Oligonucleotides**		

Primer for gentotyping: *Gpr183* E2: TGAGTCGGAGGCTAGCTTGT	This paper	N/A
Primer for gentotyping: *Gpr183* F1: GTGGCTTTAATGCTGTGGAA	This paper	N/A
Primer for gentotyping: *Gpr183* B’2R: CTTGTACTGGGTTCAACGCA	This paper	N/A
Primer for quantitative RT-PCR: *Hprt* Forward: CTGGTGAAAAGGACCTCTCG	Sigma	N/A
Primer for quantitative RT-PCR: *Hprt* Reverse: TGAAGTACTCATTATAGTCAAGGGCA	Sigma	N/A
Probe for quantitative RT-PCR: *Hprt*: [6FAM]TGTTGGATACAGGCCAGACTTTGTTGGAT[BHQ1]	Sigma	N/A
Primer for quantitative RT-PCR: *Ch25h* Forward: GCGACGCTACAAGATCCA	Yi et al., 2012	N/A
Primer for quantitative RT-PCR: *Ch25h* Reverse: CACGAACACCAGGTGCTG	Yi et al., 2012	N/A
Primer for quantitative RT-PCR: *Cyp7b1* Forward: TTCCTCCACTCATACACAATG	Yi et al., 2012	N/A
Primer for quantitative RT-PCR: *Cyp7b1* Reverse: CGTGCTTTTCTTCTTACCATC	Yi et al., 2012	N/A
Primer for quantitative RT-PCR: *Hsd3b7* Forward: ACCATCCACAAAGTCAACG	Yi et al., 2012	N/A
Primer for quantitative RT-PCR: *Hsd3b7* Reverse: TCTTCATTGCCCCTGTAGA	Yi et al., 2012	N/A

**Software and Algorithms**		

FlowJo 9 and 10	Tree Star	https://www.flowjo.com/solutions/flowjo/downloads
GraphPad Prism 6.0	GraphPad	https://www.graphpad.com
Imaris 8	Bitplane	http://www.bitplane.com/download
NIS-Elements Viewer 4.0	Nikon	https://www.nikoninstruments.com/en_EU/Products/Software/NIS-Elements-Advanced-Research/NIS-Elements-Viewer
Zeiss ZEN 2010	Zeiss	https://www.zeiss.ch/mikroskopie/downloads/zen.html

**Other**		

TaqMan Assay: Mouse *Ahr*	Life Technologies	Mm00478932_m1
TaqMan Assay: Mouse *Ch25h*	Life Technologies	Mm00515486_s1
TaqMan Assay: Mouse *Ccl20*	Life Technologies	Mm01268754_m1
TaqMan Assay: Mouse *Csf2*	Life Technologies	Mm01290062_m1
TaqMan Assay: Mouse *Cxcl13*	Life Technologies	Mm00444534_m1
TaqMan Assay: Mouse *Cyp7b1*	Life Technologies	Mm00484157_m1
TaqMan Assay: Mouse *Gpr183*	Life Technologies	Mm01234672_m1
TaqMan Assay: Mouse *Hsd3b7*	Life Technologies	Mm01159156_g1
TaqMan Assay: Mouse *Lta*	Life Technologies	Mm00440228_gH
TaqMan Assay: Mouse *Ltb*	Life Technologies	Mm00434774_g1
TaqMan Assay: Human *HPRT*	Life Technologies	Hs02800695_m1
TaqMan Assay: Human *CH25H*	Life Technologies	Hs02379634_s1
TaqMan Assay: Human *CYP7B1*	Life Technologies	Hs01046431_m1
TaqMan Assay: Human *HSD3B7*	Life Technologies	Hs00986913_g1
TaqMan Assay: Human *CXCL8*	Life Technologies	Hs00174103_m1
Quantitect primer assay: Mouse *Hprt*	QIAGEN	330001

### Contact for Reagent and Resource Sharing

Further information and requests for resources and reagents should be directed to and will be fulfilled by the Lead Contact, Tim Willinger (tim.willinger@ki.se).

### Experimental Model and Subject Details

#### Mice

All mice used were on the B6 genetic background. *Gpr183*^*GFP/+*^ mice were previously described (Pereira et al., 2009). *Gpr183*^−/−^ and *Gpr183*^flox/flox^ mice were generated at Yale University as described in Generation of *Gpr183*-deficient Mice below. *Gpr183*^−/−^ mice were bred to *Rag1*^−/−^ mice to generate *Gpr183*^−/−^ *Rag1*^−/−^ mice and *Gpr183*^+/+^ *Rag1*^−/−^ controls. *Gpr183*^−/−^ mice were also bred to *Rorc(γt)*^*GFP*^ transgenic mice (Lochner et al., 2008), provided by Dr. Eberl (Pasteur Institute), to generate *Rorc(γt)*^*GFP*^ *Gpr183*^−/−^ and *Rorc(γt)*^*GFP*^ *Gpr183*^+/+^ mice. *Gpr183*-sufficient controls for all *Gpr183*-deficient strains were co-housed littermates obtained from heterozygous x heterozygous breeding. Mice were generally used at 6-12 weeks of age and littermates of the same sex were as much as possible randomly assigned to experimental groups. All mice were maintained in individually ventilated cages under specific pathogen-free conditions at Yale University and Karolinska Institutet. Germ-free B6 mice were obtained from the Core Facility for Germ-Free Research at Karolinska Institutet. All mouse experiments were performed in accordance with protocols approved by the Institutional Animal Care and Use Committee of Yale University and the Linköping Animal Experimentation Ethics Committee.

#### Human Inflammatory Bowel Disease (IBD)

Six ulcerative colitis patients and eight controls (patients suspected to have IBD but upon colonoscopy diagnosed as non-IBD patients) were recruited at the Astrid Lindgren Children’s Hospital, Stockholm, Sweden, after signed informed consent was obtained. All patients (two females and four males) and controls (five females and three males) were untreated and had no other inflammatory or infectious diseases. RNA extraction was performed from frozen biopsies collected upon diagnostic colonoscopy, as previously described (Kvedaraite et al., 2016). Briefly, total RNA was extracted and purified from snap-frozen tissue biopsies (four 50 μm sections/patient) using the RiboPure Kit (Life Technologies). RNA was converted to cDNA using the High Capacity RNA-to-cDNA Master Mix (Applied Biosystems). cDNA was stored at −20**°**C until *CH25H*, *CYP7B1*, *HPRT*, *HSD3B7*, and *CXCL8* mRNA expression was measured with qPCR using Taqman Gene expression assays (Applied Biosystems), according to manufacturer’s protocols. The collection of patient data and colon tissue biopsies was approved by the Ethical Review Board at Karolinska Institutet (Approval 2010/32-31/4) and the investigations were conducted according to the Helsinki Declaration.

### Method Details

#### Generation of *Gpr183*-deficient Mice

Mouse genomic DNA of the *Gpr183* gene was isolated from B6 BAC clone RP24-395L16 (Children’s Hospital Oakland Research Institute). The targeting vector was constructed by PCR cloning of three genomic fragments into pEasy-FLIRT: a 5′ homology arm (2.6 kb) into NotI-BamHI sites, a floxed region containing exon 4 (3.5 kb) into the SalI site, and a 3′ homology arm (3.4 kb) into the AscI site. The final targeting construct was verified by a restriction digest and DNA sequencing. After linearization with SfiI, the targeting vector was electroporated into JM8 ES cells (B6 origin). Correctly targeted clones were identified by PCR screening, injected into blastocysts, and implanted into foster mothers. Chimeric mice were bred to B6 mice, and the F1 generation was screened for germline transmission with primers around LoxP site (primers E2: TGAGTCGGAGGCTAGCTTGT and F1: GTGGCTTTAATGCTGTGGAA). For the generation of *Gpr183*^−/−^ mice, mice with germline transmission were bred to *Tetracycline* (*Tet*)*-cre* transgenic mice (JAX), which results in deletion of the of LoxP-flanked *Gpr183* locus and *neomycin* (*neo*) gene because of recombinase activity in the female germline. Alternatively, the *neo* gene was removed by breeding to *flippase* recombinase transgenic mice (JAX), followed by breeding to *Rorc-cre* transgenic mice (Eberl and Littman, 2004) in order to generate *Rorc-cre Gpr183*^flox/flox^ mice. Genomic DNA was isolated from tail or ear biopsies. Wild-type, floxed, and deleted *Gpr183* alleles were distinguished with primers E2, F1, and B’2R (CTTGTACTGGGTTCAACGCA).

#### Bone Marrow Chimeras

Irradiated (2x500 cGy) *Gpr183*^+/+^, *Gpr183*^−/−^, *Ch25h* ^+/+^, or *Ch25h* ^−/−^ recipient mice received 2x10^6^ bone marrow cells from the indicated donor mice by intravenous (i.v.) injection. To generate mixed bone marrow chimeras, bone marrow cells from *Gpr183*^+/+^ or *Gpr183*^−/−^ mice were mixed at a ratio of 9:1 with B6 cells. 2x10^6^ cell mixture was injected i.v. into irradiated *Rag1*^−/−^ (1x600 cGy) or B6 (2x500 cGy) recipients. Chimeric mice were kept on prophylactic antibiotics (Sulfatrim) for 3 weeks and analyzed 8-12 weeks after reconstitution.

#### Immunofluorescence Microscopy

Colons (without cecum) and small intestines were fixed in 1%–4% PFA, dehydrated in a 10%→20%→30% sucrose gradient, and frozen in OCT medium. 7-8 μm sections were cut and stained with the following Abs (all from Biolegend): CD11c (N418)-APC, CD45.1 (A20)-AF488, CD45.2 (104)-AF647, CD90.1 (OX7)-AF488, CD90.2 (30-H12)-AF647, B220 (RA3-6B2)-APC. CD34 (RAM34)-eFluor660 was from eBioscience and Syrian hamster Ab to Podoplanin was a gift from A. Farr (University of Washington, Seattle). Cy3-conjugated α-Syrian hamster IgG was from Jackson Immunoresearch. GFP expression in *Gpr183*^*GFP/+*^ and *Rorc(γt)*^*GFP*^ mice was visualized with AF488-conjugated α-GFP Ab (Invitrogen). Alternatively, RORγt immunofluorescence was examined with α-RORγt (AFKJS-9) Ab using a previously published protocol (Mackley et al., 2015). Briefly, after cell permeabilization with saponin and various blocking steps, sections were stained with primary rat α-RORγt (AFKJS-9) Ab, followed by signal amplification with secondary (FITC-conjugated donkey α-rat secondary Ab), tertiary (Alexa Fluor 488-conjugated rabbit α-fluorescein Ab), and quaternary (Alexa Fluor 488-conjugated donkey α-rabbit Ab) Abs. Finally, sections were co-stained with APC-conjugated α-B220 Ab. GATA3 immunofluorescence was determined using the same protocol with rat α-GATA3 (TWAJ) Ab and rat IgG2a isotype was used as a control. KLRG1 was detected with hamster α-KLRG1 (2F1) Ab and visualized with AF647-conjugated goat α-hamster IgG (ThermoFisher). Hamster IgG2a isotype was used as a control for KLRG1 staining. Images were acquired on a Nikon A1R confocal microscope and processed with NIS-Elements Imaging software.

#### Quantification of Intestinal CPs and ILFs

CPs and ILFs were quantified in the following gene-deficient strains:

**Table:** 

Strain	Control	Breeding	RORγt detection
*Rorc(γt)*^GFP^*Gpr183*^−/−^	*Rorc(γt)*^GFP^*Gpr183*^+/+^	*Rorc(γt)*^GFP^*Gpr183*^+/−^ x *Gpr183*^+/−^	RORγt-GFP
*Gpr183*^−/−^*Rag1*^−/−^	*Gpr183*^+/+^*Rag1*^−/−^	*Gpr183*^+/−^*Rag1*^−/−^ x *Gpr183*^+/−^*Rag1*^−/−^	RORγt Ab
*Rorc-cre Gpr183*^flox/flox^	*Gpr183*^flox/flox^ or *Gpr183*^flox/+^	*Rorc-cre Gpr183*^flox/+^ x *Gpr183*^flox/flox^	RORγt Ab
*Ch25h*^−/−^	*Ch25h*^+/+^	*Ch25h*^+/+^ x *Ch25h*^+/+^ *Ch25h*^−/−^ x *Ch25h*^−/−^	RORγt Ab

(With the exception of *Ch25h*^+/+^ and *Ch25h*^−/−^ mice, all controls were co-housed littermates.)

Colon (without cecum) and distal small intestine of 6 cm length were fixed and sectioned. Tissue sections were stained for RORγt and B220 to visualize CPs (RORγt^+^B220^-^ clusters) and ILFs (RORγt^+^B220^+^ clusters). RORγt was detected either with AF488-conjugated α-GFP Ab in *Rorc(γt)*^GFP^ *Gpr18*3^+/+^ and *Rorc(γt)*^GFP^ *Gpr183*^−/−^ mice or with α-RORγt Ab in all other strains of mice (Mackley et al., 2015) – see Immunofluorescence Microscopy above. CPs and ILFs were counted in 20 sections taken at 80 μM intervals through the whole depth of the tissue, with the exception of colonic CPs and ILFs in *Rorc(γt)*^GFP^ *Gpr18*3^+/+^ and *Rorc(γt)*^GFP^ *Gpr183*^−/−^ mice, where 30 sections were counted (Figure 2B).

#### Isolation of Immune Cells and Flow Cytometry

Spleens and lymph nodes were mechanically disrupted and filtered through 100 μm nylon mesh. Lamina propria lymphocytes (LPLs) were isolated from the colon (incl. cecum) and small intestine using a standard protocol. Dissected intestines were flushed with cold PBS to remove feces and mucus, opened longitudinally, and cut into ∼1cm pieces. To remove epithelial cells, intestinal pieces were incubated twice in HBSS supplemented with 5% FCS, 10mM HEPES, and 5mM EDTA for 15 minutes (min) at 37°C (shaking). After each incubation, tubes were vortexed and the supernatant, containing epithelial cells, discarded. Pieces were then washed with RPMI 1640 medium supplemented with 10% FCS, 2mM L-Glutamine, 1% penicillin-streptomycin, and 55 μM β-mercaptoethanol (R10 medium), minced with scissors, and incubated in digestion medium (HBSS containing 5% FCS, 0.2mg/ml collagenase IV, and 0.1mg/ml DNase I) for 20 min at 37°C (shaking). After vortexing, supernatants were collected, and passed sequentially through 100 μm and 70 μm cell strainers. Digestion was repeated twice and the supernatants pooled. LPLs were then isolated with a 40%/80% Percoll gradient and washed for FACS analysis. Single-cell suspensions were stained with fluorochrome-labeled Abs (see Key Resources Table) for 20-30 min on ice. After surface staining, cells were stained with fixable viability dye-eFluor 506 (eBioscience) according to the manufacturer’s instructions. Intracellular staining for RORγt (B2D)-PE-eFluor610 (eBioscience) was performed with the FoxP3 transcription factor buffer set (eBioscience). Alternatively, *Rorc(γt)*^*GFP*^ transgenic mice were used to detect RORγt. Cells were acquired on a LSRII Fortessa flow cytometer (BD Biosciences) and analyzed with FlowJoV10 software.

#### LTα_1_β_2_ Cell Surface Expression

Surface LTα_1_β_2_ was detected using a LTβR-Fc chimera (R&D Systems) as described (Bando et al., 2015). Briefly, cells were blocked with 40 μg/ml donkey α-mouse Fab fragments (Jackson ImmunoResearch) for 20 min on ice before staining with 1 μg/ml LTβR-Fc chimera for 30 min on ice. After washing, cells were incubated for 20 min on ice with 6.5 μg/ml biotinylated donkey α-mouse IgG (Jackson ImmunoResearch). Cells were then washed, blocked with 2% mouse/rat serum, and stained with surface Ab mix including PE-conjugated streptavidin.

#### Intracellular Cytokine Staining

For IL-22 staining, LPLs were stimulated with 40ng/ml of recombinant IL-23 (R&D Systems) for 3 hr together with Golgi Stop (BD Biosciences). For IL-5, IL-17F and IL-17A staining, LPLs were stimulated with 100ng/ml phorbol-12-myristate 13-acetate (PMA) and 1 μg/ml Ionomycin for 3 hr in the presence of Golgi Stop at 37C, 5% CO2. Alternatively, single cell suspensions from mesenteric lymph nodes, small intestine and colon were stimulated with 20ng/ml IL-1β, 20ng/ml IL-23 and a commercially available cell stimulation cocktail (ebioscience) containing PMA, Ionomycin, Brefeldin A and Monensin in the absence (DMSO) or presence of 10nM 7α,25-OHC (Avanti Polar Lipids) for 4 hr. After stimulation, cells were first surface-stained and subsequently incubated with Fixation/Permeabilization buffer (Foxp3 Transcription Factor Staining Buffer Kit, eBioscience) at 4°C for 30 min. For intracellular transcription factor and cytokine staining after fixation, cells were incubated for 20 min at room temperature with fluorochrome-conjugated Abs (see Key Resources Table).

#### Cell Sorting

Cells were isolated from the colon and small intestine as previously described (Villablanca et al., 2011) with modifications. Colon and small intestine sections were washed in PBS, cut into 1cm-pieces, and incubated with 20mL of HBSS containing 5% FCS, 5mM EDTA, 1mM DTT, and 15mM HEPES at 37°C for 30 min on a shaking incubator. The supernatant, i.e., the intestinal epithelial cell (IEC) fraction, was saved and the pieces were subsequently washed and incubated with 10mL of serum-free HBSS containing 0.15mg/mL Liberase TL (Roche) and 0.1mg/mL DNase I at 37°C for 45 min at 600rpm. The resulting lamina propria (LP) cell suspension was then passed through a 100 μm nylon mesh and centrifuged. For isolation of epithelial (CD45^-^Epcam^+^), CD45^-^Epcam^-^, and CD45^+^ cells (Figure 6B, upper panel), the IEC and LP fractions were mixed, centrifuged and stained. For isolation of ILC3s, the LP fraction was further processed for Percoll gradient separation (44%/67%, Sigma) at 600 × g for 20 min, washed and stained. In Figure 4D and Figure S6E, ILC3s were sorted from *Rag1*-deficient *Rorc(γt)*^*GFP*^ *Gpr18*3^+/+^ and *Gpr183*^−/−^ mice and were defined as DAPI^-^CD45^+^CD11c^−^CD90.2^+^KLRG1^−^RORγt-GFP^+^ cells. In Figure 6B (middle panel), cells were sorted from B6 mice as B cells (CD45^+^B220^+^MHC class II^+^), LTi-like ILC3s (CD45^+^Lin(CD3/CD5/B220/CD11c/NK1.1) ^−^CD127^+^CD90^+^KLRG1^−^CCR6^+^), DCs (CD45^+^CD11c^+^Class II^+^), and CD45^−^Epcam^-^FSC^hi^ cells. Cell populations were sorted using BD FACSAriaI (BD Biosciences).

#### Isolation of Stromal Cells

Colonic stromal cell subsets (Figure 6B, lower panel) were isolated by cell sorting as described (Stzepourginski et al., 2017). CXCL13^+^ stromal cells (Figures S7F and G) were isolated from *Cxcl13*-EYFP reporter mice (Onder et al., 2017) as follows: Colonic tissue was harvested and incubated three times for 15 min at room temperature under constant agitation with PBS containing 5% FCS (Lonza), 5mM EDTA (Sigma), 10mM HEPES (Sigma) and 1mM DTT (PD buffer) in order to dissociate the epithelial layer. The tissue was subsequently washed with HBSS containing 10mM HEPES and digested three times for 20 min at 37°C under constant agitation with 120mg/ml collagenase P (Roche), 25mg/ml DNase I (Applichem) and 5μg/ml Dispase I (Roche) in RPMI 1640. To enrich the fraction of stromal cells, suspensions were first purified with a 30% Percoll gradient for 20 min at 1,800rpm and 4°C. Then hematopoietic cells and erythrocytes were depleted by incubating the cell suspension with MACS α-CD45 and α-Ter119 microbeads and passing through a MACS LS column (Miltenyi Biotec). Unbound single cell suspensions were used for further flow cytometric analysis with α-CD31 (390)-PerCP/Cy5.5 and α-Podoplanin (8.1.1)-PE Abs (Biolegend) and cell sorting was performed via Bio-Rad S3 cell sorter.

#### Chemotaxis Assays

Splenocytes or LPLs from the colon were rested in migration medium (RPMI 1640/0.5% fatty acid-free BSA (Sigma)/10mM HEPES) for 30 min at 37°C/5% CO_2_. Migration of 1-2x10^6^ input cells through 5 μM Transwells (Corning) in response to the indicated concentrations of 7α,25-OHC (Avanti Polar Lipids) was assessed after 3-4 hr. Cells in the bottom chamber were stained with fluorochrome-conjugated Abs to identify LTi-like ILC3s (CD45^+^CD11b^−^CD11c^−^Gr-1^−^CD90.2^+^CD127^+^NK1.1^−^) by flow cytometry (Figure 1D). In Figure 1E, *Rag1*-deficient *Rorc(γt)*^*GFP*^ transgenic mice were used and the following cell populations identified: NK cells (B220^−^CD3^−^CD5^−^CD11c^−^RORγt-GFP^−^NK1.1^+^); ILC2s (B220^−^CD3^−^CD5^−^CD11c^−^RORγt-GFP^−^NK1.1^−^KLRG1^+^); CCR6^−^ ILC3s (B220^−^CD3^−^CD5^−^CD11c^−^RORγt-GFP^+^CCR6^−^); CD4^+^ LTi-like ILC3s (B220^−^CD3^−^CD5^−^CD11c^−^RORγt-GFP^+^CCR6^+^CD117^+^CD4^+^). For B cell (TCRβ^−^CD4^−^CD11b^−^CD11c^−^Ly6G^−^B220^+^) chemotaxis B6 mice were used. In Figure 1F, NK cells (B220^−^CD11c^−^CD127^−^NK1.1^+^), ILC2s (B220^−^CD11c^−^CD127^+^CD90.2^+^NK1.1^−^KLRG1^+^), and ILC3s (B220^−^CD11c^−^CD127^+^CD90.2^+^NK1.1^−^KLRG1^−^CD45^mid^) were identified in Rag1^−/−^ mice. The chemotactic response was measured either as frequency of cells compared to input (Figure 1D) or as relative migration, i.e., number of cells migrated toward 7α,25-OHC compared to medium (Figures 1E and 1F). To measure GPR183 ligand activity, the chemotaxis of the M12 B cell line transduced with GPR183-GFP toward colon homogenates was examined as described (Kelly et al., 2011).

#### IgA Production

Co-housed *Gpr18*3^+/+^ and *Gpr183*^−/−^ mice littermates were either left untreated or received a total of five intraperitoneal (i.p.) injections of 100 μg α-TCRβ (H57-597) Ab (Bio X Cell) every three days. Three days after the last injection, sera and feces were collected. IgA concentrations were determined in homogenized feces and sera by sandwich ELISA using goat α-mouse IgA alpha chain Abs (Abcam). Mouse IgA kappa (Abcam) was used as a standard.

#### Quantitative RT-PCR

Total RNA was extracted from colon tissue (∼1cm piece) or purified cells with either TRIzol reagent (Invitrogen) or RNeasy Micro Kit (QIAGEN) and used for cDNA synthesis with the SuperScript First-Strand Synthesis System (Invitrogen), iScript cDNA Synthesis Kit (BioRad), or High-Capacity cDNA Reverse Transcriptase Kit (Applied Biosystems). Quantitative RT-PCR was performed on a 7500 Fast Real-Time PCR system or PCRQuant Studio 5 Real-Time PCR system (Applied Biosystems) with primer-probe sets purchased from Applied Biosystems (*Ahr*, *Ch25h*, *Ccl20*, *Csf2*, *Cxcl13*, *Cyp7b1*, *Gpr183*, *Hsd3b7*, *Lta*, *Ltb*) or Sigma (*Hprt*). Alternatively, quantitative PCR was performed using Light Cycler 480 SYBR Green I Master mix (Roche Diagnosis) on a LightCycler 480 II machine (Roche Diagnosis). Expression levels were measured using following primers (Yi et al., 2012): *Ch25h* Fw-GCGACGCTACAAGATCCA, Rv-CACGAACACCAGGTGCTG; *Cyp7b1* Fw-TTCCTCCACTCATACACAATG, Rv-CGTGCTTTTCTTCTTACCATC; *Hsd3b7* Fw-ACCATCCACAAAGTCAACG, Rv-TCTTCATTGCCCCTGTAGA; and QIAGEN Quantitect primer for *Hprt* (ENSMUSG00000025630).

#### Gene Expression in Colonic Lymphoid Structures

Intestines from human *CD2*^*GFP*^ transgenic mice (de Boer et al., 2003) were flushed with cold PBS (GIBCO) and opened longitudinally. Mucus and epithelium were scraped mechanically using 1.5 mm coverslips (Thermo Scientific). Under a fluorescence Stereo Lumar Zeiss V12 microscope, intestines were imaged with a NeoLumar S 0.8x objective using the GFP filter. CPs, ILFs, colonic patches, and Peyer’s patches were dissected using a surgical blade (Swann-Morton). Tissues were collected in cold RPMI supplemented with 1% HEPES, sodium pyruvate, glutamine, streptomycin and penicillin and 0.1% β-mercaptoethanol (GIBCO). After tissue dissection, supplemented media was removed and tissues were snap-frozen using liquid nitrogen and dry ice for later RNA extraction.

#### Colon Histology *Cxcl13*-EYFP Reporter Mice

Colons were fixed with 4% paraformaldehyde (Merck) in PBS under agitation at 4°C. Fixed colons were further washed with PBS containing 1% Triton X-100 (Sigma) and 2% FCS (Sigma) overnight at 4°C. Samples were further processed with FIX&PERM cell fixation & permeabilization kit (Thermo Fischer) for RORγt staining. Briefly, samples were incubated with 1x fixation/permeabilization buffer overnight. After washing with permeabilization buffer, samples were stained with rat α-RORγt (AFKJS-9) Ab (eBioscience) for 2 hr at 4°C. Samples were then stained with biotin conjugated α-rat secondary Ab overnight after washing with permeabilization buffer. Finally, samples were incubated with Cy3-conjugated streptavidin (Jackson ImmunoResearch) and AF647-conjugated α-B220 (RA3-6B2) Ab (Biolegend) for 2 hr. Podoplanin was detected with 8.1.1 Ab (Biolegend). Microscopy analysis was performed using a confocal microscope (Zeiss LSM-710) and images were processed with ZEN 2010 software (Carl Zeiss) and Imaris (Bitplane).

#### Colitis Model

Colitis was induced by i.p. injection of 100 μg CD40 (FGK45) Ab (Bio X Cell) into mice on a *Rag1*-deficient background as described (Uhlig et al., 2006). At the indicated time points, proximal colons were harvested for RNA extraction, chemotaxis assay, immunofluorescence microscopy, or fixed in 3.7% formalin for histological analysis. Gut inflammation was assessed in paraffin-embedded colon sections stained with H&E. Inflammatory foci were identified as clusters of leukocytes near/at the tip of colonic folds in the proximal colon as described (Uhlig et al., 2006). They were distinguished from CPs by their distinct location because the latter are found at the base of the colonic folds near the crypts. Colitis score was calculated using a semiquantitative criterion-based method (score 0–6) as described (Huber et al., 2004). Colon sections were scored in a blinded manner.

### Quantification and Statistical Analysis

Statistical parameters including number of biological replicates and repeat experiments, data dispersion and precision measures (mean and SEM) and p values for statistical significance (α = 0.05) are reported in Figures and Figure Legends. Student’s t test was used to determine statistical significance between two groups. For multigroup comparisons, we applied one-way ANOVA with post hoc testing using Tukey’s Multiple Comparison Test. Asterisk coding is indicated in Figure Legends as ^∗^, p < 0.05; ^∗∗^, p < 0.01; ^∗∗∗^, p < 0.001; ^∗∗∗∗^, p < 0.0001. Statistical analysis was performed using GraphPad Prism 6.

## Supplemental Information

Document S1. Figures S1–S7

Document S2. Article plus Supplemental Information
