# Supplementary material for: Oxysterol Sensing through the Receptor GPR183 Promotes the Lymphoid-Tissue-Inducing Function of Innate Lymphoid Cells and Colonic Inflammation
Source: Immunity. 2018 Jan 16;48(1):120–132.e8. doi: 10.1016/j.immuni.2017.11.020 (PMC5772175; doi:10.1016/j.immuni.2017.11.020)
Supplement: Document S1. Figures S1–S7 [file mmc1.pdf]

Immunity, Volume *48*

Supplemental Information

Oxysterol Sensing through the Receptor GPR183 Promotes the Lymphoid-Tissue-Inducing Function of Innate Lymphoid Cells and Colonic Inflammation

Johanna Emgård, Hana Kammoun, Bethania García-Cassani, Julie Chesné, Sara M. Parigi, Jean-Marie Jacob, Hung-Wei Cheng, Elza Evren, Srustidhar Das, Paulo Czarnewski, Natalie Sleiers, Felipe Melo-Gonzalez, Egle Kvedaraite, Mattias Svensson, Elke Scandella, Matthew R. Hepworth, Samuel Huber, Burkhard Ludewig, Lucie Peduto, Eduardo J. Villablanca, Henrique Veiga-Fernandes, João P. Pereira, Richard A. Flavell, and Tim Willinger

Figure S1

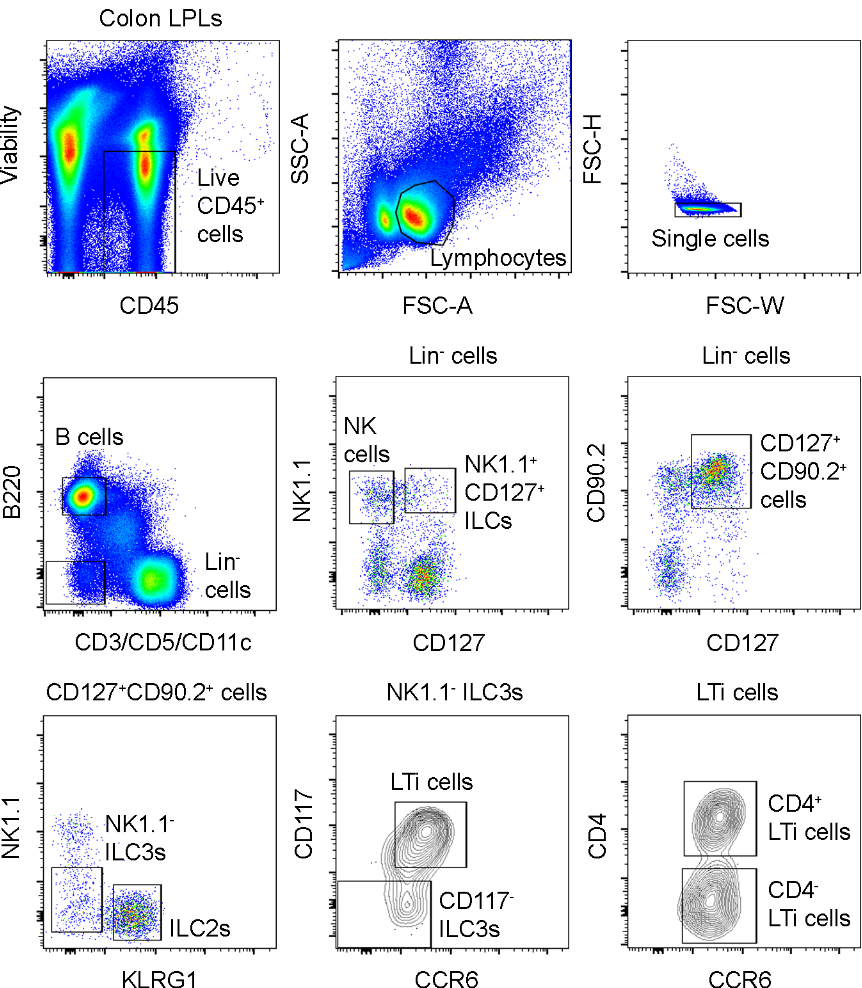

Colon LPLs
Viability
CD45
Live CD45+ cells
SSC-A
FSC-A
Lymphocytes
FSC-H
FSC-W
Single cells
B220
CD3/CD5/CD11c
B cells
Lin- cells
Lin- cells
NK1.1
CD127
NK cells
NK1.1+ CD127+ ILCs
Lin- cells
CD90.2
CD127
CD127+ CD90.2+ cells
CD127+CD90.2+ cells
NK1.1
KLRG1
NK1.1- ILC3s
ILC2s
NK1.1- ILC3s
CD117
CCR6
LTi cells
CD117- ILC3s
LTi cells
CD4
CCR6
CD4+ LTi cells
CD4- LTi cells

Figure S1, related to Figure 1: Gating strategy for analyzing GPR183-GFP expression in colonic ILC subsets.

Lamina propria lymphocytes (LPLs) were isolated from the colon (incl. cecum) of *Gpr183*$^{GFP/+}$ mice. After gating on live CD45$^+$ single lymphocytes, Lineage-negative cells (Lin$^-$) were gated as B220$^-$CD3$^-$CD5$^-$CD11c$^-$ cells and B lymphocytes as B220$^+$CD3$^-$CD5$^-$CD11c$^-$ cells. ILC subsets were identified as indicated: (1) NK cells (Lin$^-$NK1.1$^+$CD127$^-$); (2) NK1.1$^+$CD127$^+$ ILCs (Lin$^-$NK1.1$^+$CD127$^+$); (3) ILC2s (Lin$^-$CD127$^+$CD90.2$^+$NK1.1$^-$KLRG1$^+$); (4) NK1.1$^-$ ILC3s (Lin$^-$CD127$^+$CD90.2$^+$NK1.1$^-$KLRG1$^-$). NK1.1$^-$ ILC3s were then divided into CD117$^-$CCR6$^-$ and LTi-like (CD117$^+$CCR6$^+$) subsets. The latter includes CD4$^+$ and CD4$^-$ LTi-like ILC3s. Data are representative of three experiments.

Figure S2

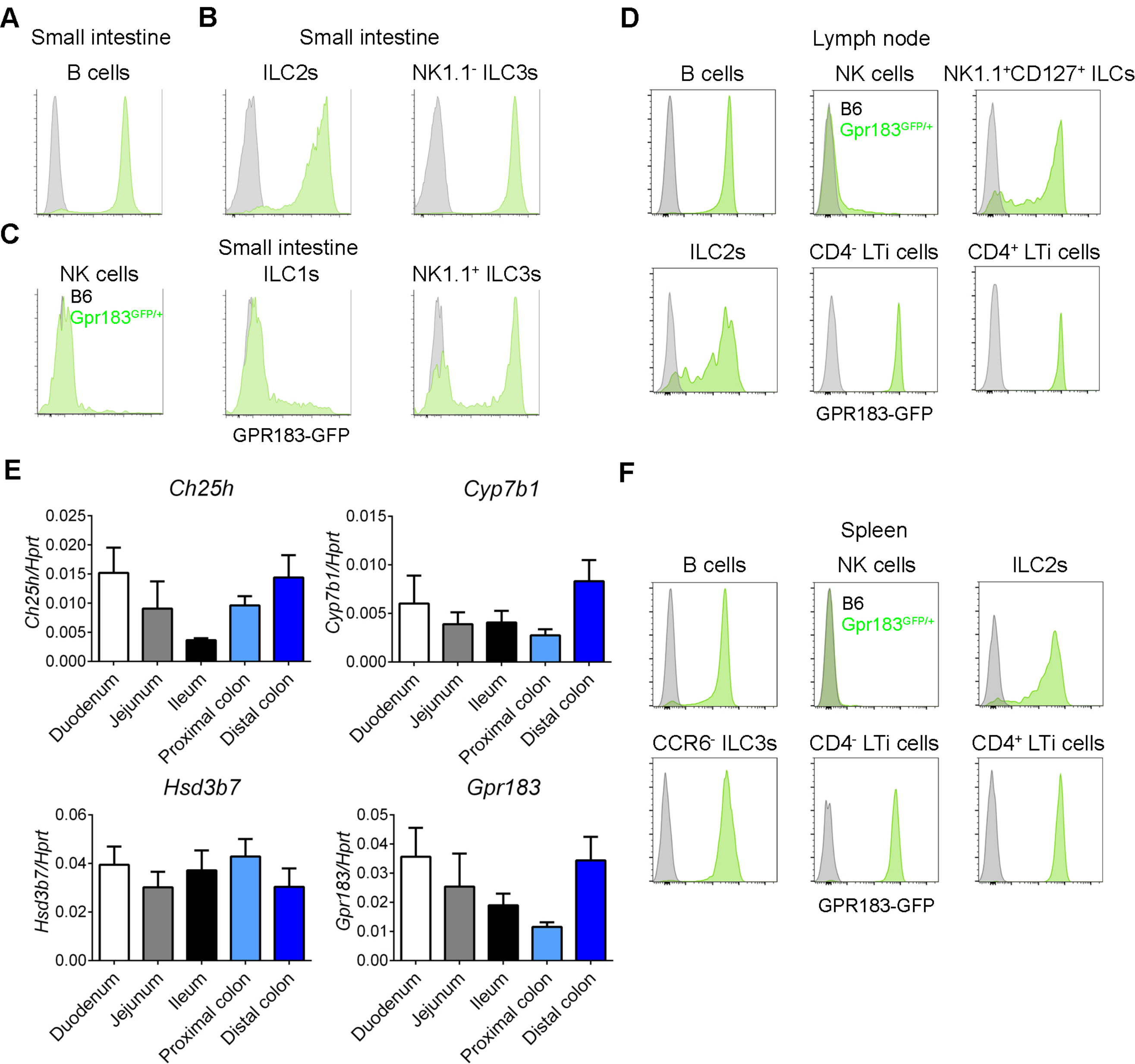
A
Small intestine
B cells
B
Small intestine
ILC2s
NK1.1⁻ ILC3s
C
NK cells
B6
Gpr183GFP/+
Small intestine
ILC1s
NK1.1⁺ ILC3s
GPR183-GFP
D
Lymph node
B cells
NK cells
B6
Gpr183GFP/+
NK1.1⁺CD127⁺ ILCs
ILC2s
CD4⁻ LTi cells
CD4⁺ LTi cells
GPR183-GFP
E
Ch25h
Ch25h/Hprt
0.025
0.020
0.015
0.010
0.005
0.000
Cyp7b1
Cyp7b1/Hprt
0.015
0.010
0.005
0.000
Duodenum
Jejunum
Ileum
Proximal colon
Distal colon
Hsd3b7
Hsd3b7/Hprt
0.06
0.04
0.02
0.00
Gpr183
Gpr183/Hprt
0.05
0.04
0.03
0.02
0.01
0.00
F
Spleen
B cells
NK cells
B6
Gpr183GFP/+
ILC2s
CCR6⁻ ILC3s
CD4⁻ LTi cells
CD4⁺ LTi cells
GPR183-GFP

Figure S2, related to Figure 1: GPR183-GFP expression in small intestine, lymph node, spleen and expression of GR183 ligand-generating enzymes in the small intestine.

(A-C) GFP expression in lamina propria B cells (A) and ILC subsets (B and C) from the small intestine of $Gpr183^{GFP/+}$ reporter (green histograms) and B6 control mice (grey histograms). ILC subsets (live $CD45^+Lin^-$ lymphocytes) were gated as: (1) NK cells ($NK1.1^+NKp46^+CD127^-CD27^+$); (2) ILC1s ($NK1.1^+NKp46^+CD127^+CD27^+$); (3) ILC2s ($CD127^+CD90.2^+NK1.1^-KLRG1^+$); (4) $NK1.1^+$ ILC3s ($NK1.1^+NKp46^+CD127^+CD27^-$); (5) $NK1.1^-$ ILC3s ($CD127^+CD90.2^+NK1.1^-KLRG1^-$). (D) GFP expression in B cells and ILC subsets from lymph node of $Gpr183^{GFP/+}$ reporter (green histograms) and B6 control mice (grey histograms). (E) *Ch25h*, *Cyp7b1*, *Hsd3b7*, and *Gpr183* mRNA expression in the indicated regions from the small and large intestine of B6 mice (*n* =8). mRNA expression was normalized to *Hprt*. Data are represented as mean ± SEM. (F) GFP expression in B cells and ILC subsets from spleen of $Gpr183^{GFP/+}$ reporter (green histograms) and B6 control mice (grey histograms). Data are representative of or combined from two (A, C, E) or three (B, D, F) experiments.

Figure S3

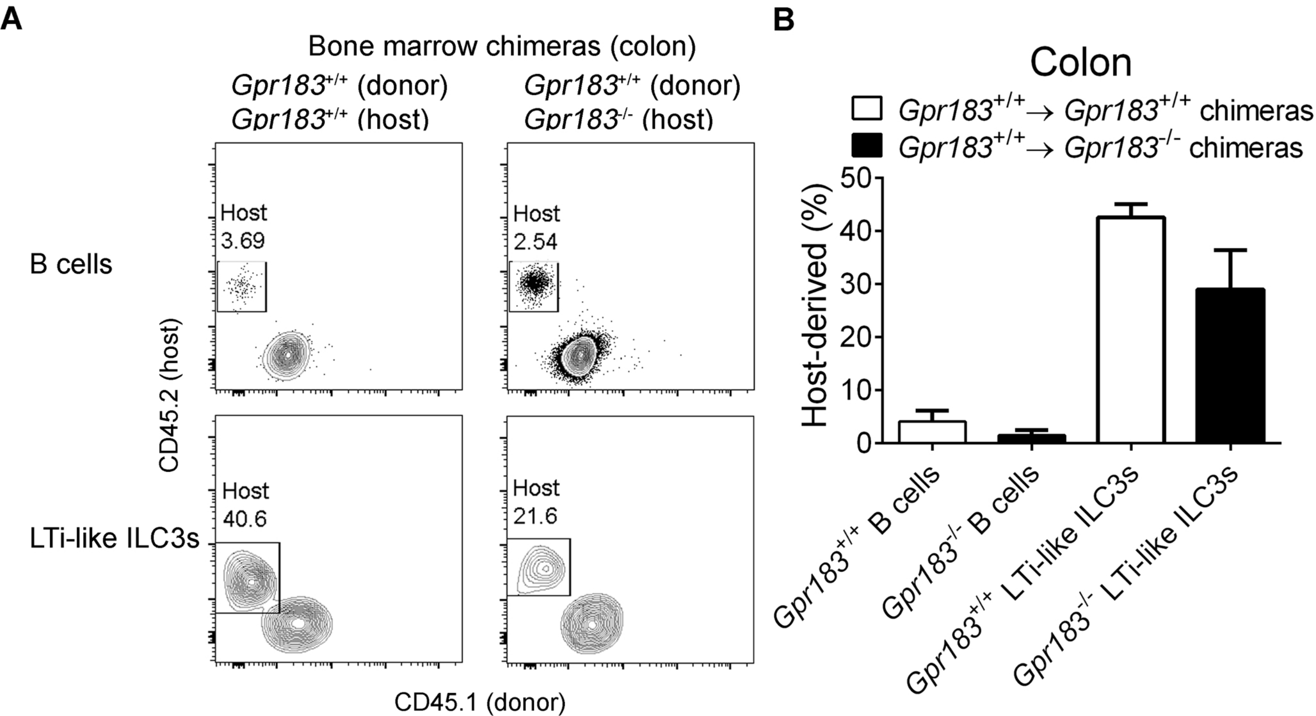
A
Bone marrow chimeras (colon)
Gpr183+/+ (donor)
Gpr183+/+ (host)
Gpr183+/+ (donor)
Gpr183-/- (host)
B cells
LTi-like ILC3s
Host
3.69
Host
2.54
Host
40.6
Host
21.6
CD45.2 (host)
CD45.1 (donor)
B
Colon
Gpr183+/+→ Gpr183+/+ chimeras
Gpr183+/+→ Gpr183-/- chimeras
Host-derived (%)
50
40
30
20
10
0
Gpr183+/+ B cells
Gpr183-/- B cells
Gpr183+/+ LTi-like ILC3s
Gpr183-/- LTi-like ILC3s

Figure S3, related to Figure 2: Partial radioresistance of host LTi-like ILC3s in bone marrow chimeras.

(A and B) B cell and LTi-like ILC3 chimerism in the colon of radiation bone marrow chimeras. Bone marrow cells from B6 mice ($CD45.1^{+}$) were injected into irradiated (2x500 cGy) *Gpr183*$^{+/+}$ or *Gpr183*$^{-/-}$ recipients ($CD45.2^{+}$) to generate bone marrow chimeras. 2-3 months after reconstitution, the number of colonic SILTs was determined (see Figure 2D) and hematopoietic chimerism assessed by flow cytometry (n =2-3). Host-derived B cells and LTi-like ILC3s were identified as $CD45.2^{+}$ cells. Data are represented as mean ± SEM. Data are from two experiments.

Figure S4

A

Rag1$^{-/-}$ mice (colon)

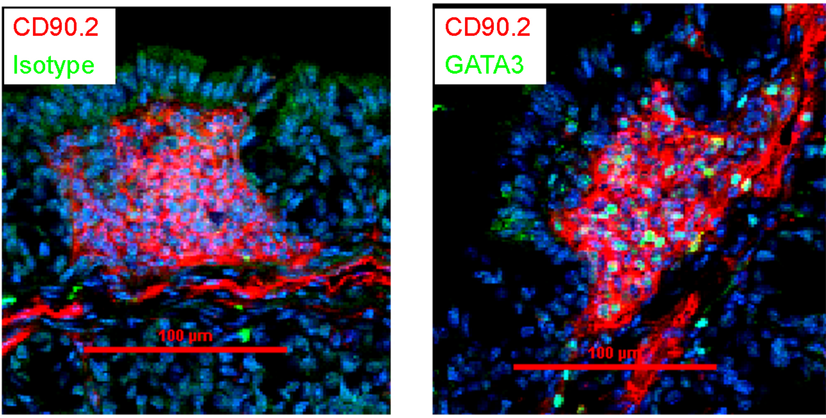

B

Rorc(γt)^{GFP} *Rag1*$^{-/-}$ mice (colon)

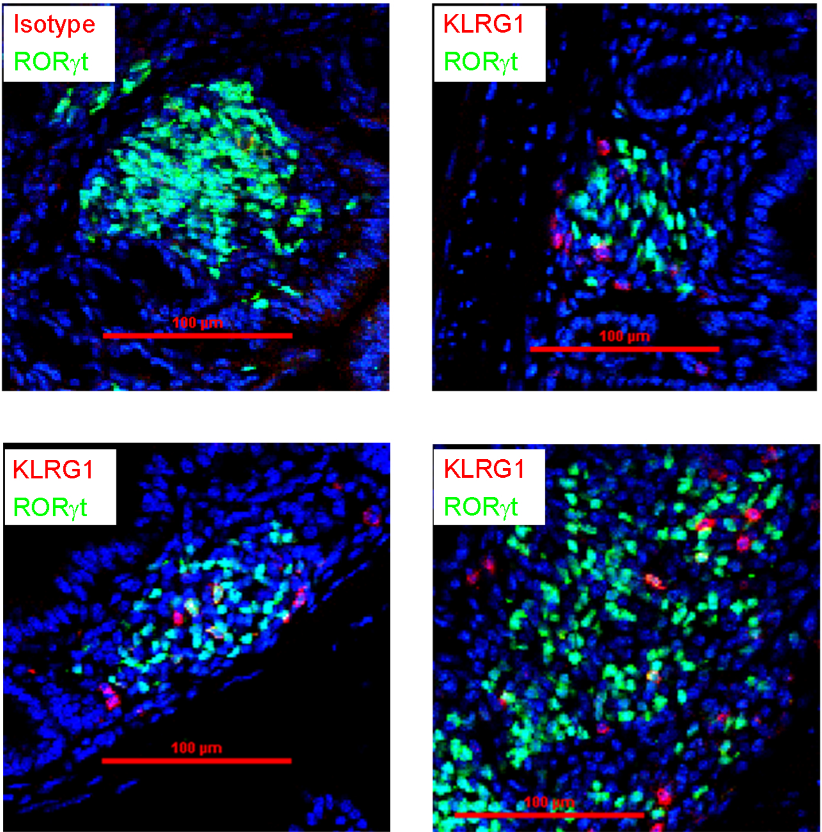

Figure S4, related to Figure 2: Localization of ILC2s in the colon.

(A) Colon sections from *Rag1*$^{-/-}$ mice were stained with α-GATA3 or isotype control Ab (green) and co-stained with Ab against CD90.2 (red). (B) Colon sections from *Rorc(γt)*^{GFP} *Rag1*$^{-/-}$ mice were stained with α-KLRG1 or isotype control Ab (red) and co-stained with Ab against GFP (green). Nuclei are in blue (DAPI). Scale bars (red) are 100 μm. Data are from one experiment.

Figure S5

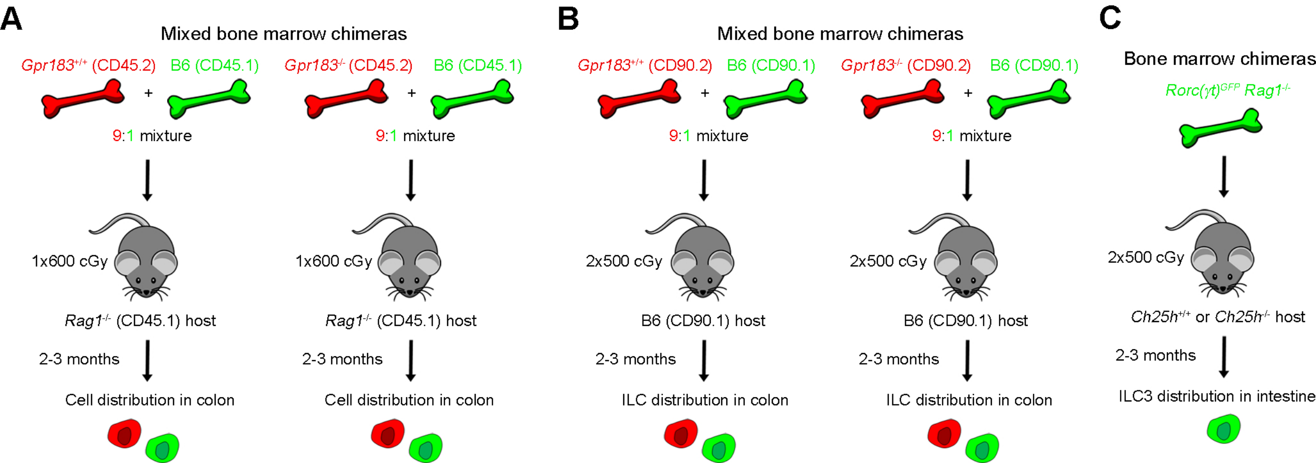

A
Mixed bone marrow chimeras
Gpr183+/+ (CD45.2)
B6 (CD45.1)
9:1 mixture
1x600 cGy
Rag1-/- (CD45.1) host
2-3 months
Cell distribution in colon
Gpr183-/- (CD45.2)
B6 (CD45.1)
9:1 mixture
1x600 cGy
Rag1-/- (CD45.1) host
2-3 months
Cell distribution in colon
B
Mixed bone marrow chimeras
Gpr183+/+ (CD90.2)
B6 (CD90.1)
9:1 mixture
2x500 cGy
B6 (CD90.1) host
2-3 months
ILC distribution in colon
Gpr183-/- (CD90.2)
B6 (CD90.1)
9:1 mixture
2x500 cGy
B6 (CD90.1) host
2-3 months
ILC distribution in colon
C
Bone marrow chimeras
Rorc(γt)GFP Rag1-/-
2x500 cGy
Ch25h+/+ or Ch25h-/- host
2-3 months
ILC3 distribution in intestine

Figure S5, related to Figure 3: Generation of bone marrow chimeras to assess intestinal ILC positioning in the absence of GPR183 or 7α,25-OHC.

(A) Mixed bone marrow chimeras to determine hematopoietic cell localization. A 9:1 mixture of either *Gpr183*$^{+/+}$ (CD45.2^{+})-B6 (CD45.1^{+}) or *Gpr183*$^{-/-}$ (CD45.2^{+})-B6 (CD45.1^{+}) bone marrow cells was injected into irradiated *Rag1*$^{-/-}$ recipients (CD45.1^{+}). Cell distribution in the colon was determined 2-3 months after transplantation (see Figure 3A). (B) Mixed bone marrow chimeras to examine ILC distribution. Bone marrow chimeras were generated as in (A), using CD90.1-CD90.2 instead of CD45.1-CD45.2 as congenic markers. Localization of *Gpr183*$^{+/+}$ and *Gpr183*$^{-/-}$ ILCs was determined as shown in Figure 3B. (C) Bone marrow chimeras to assess ILC3 localization in mice lacking the GPR183 ligand 7α,25-OHC. Bone marrow cells from *Rag1*-deficient *Rorc(*γ*t)*^{GFP} transgenic mice were injected into irradiated *Ch25h*$^{+/+}$ and *Ch25h*$^{-/-}$ recipients to generate bone marrow chimeras. ILC3 distribution in colon (see Figure 3C) and small intestine (see Figure 3D) was determined 2-3 months after transplantation.

Figure S6

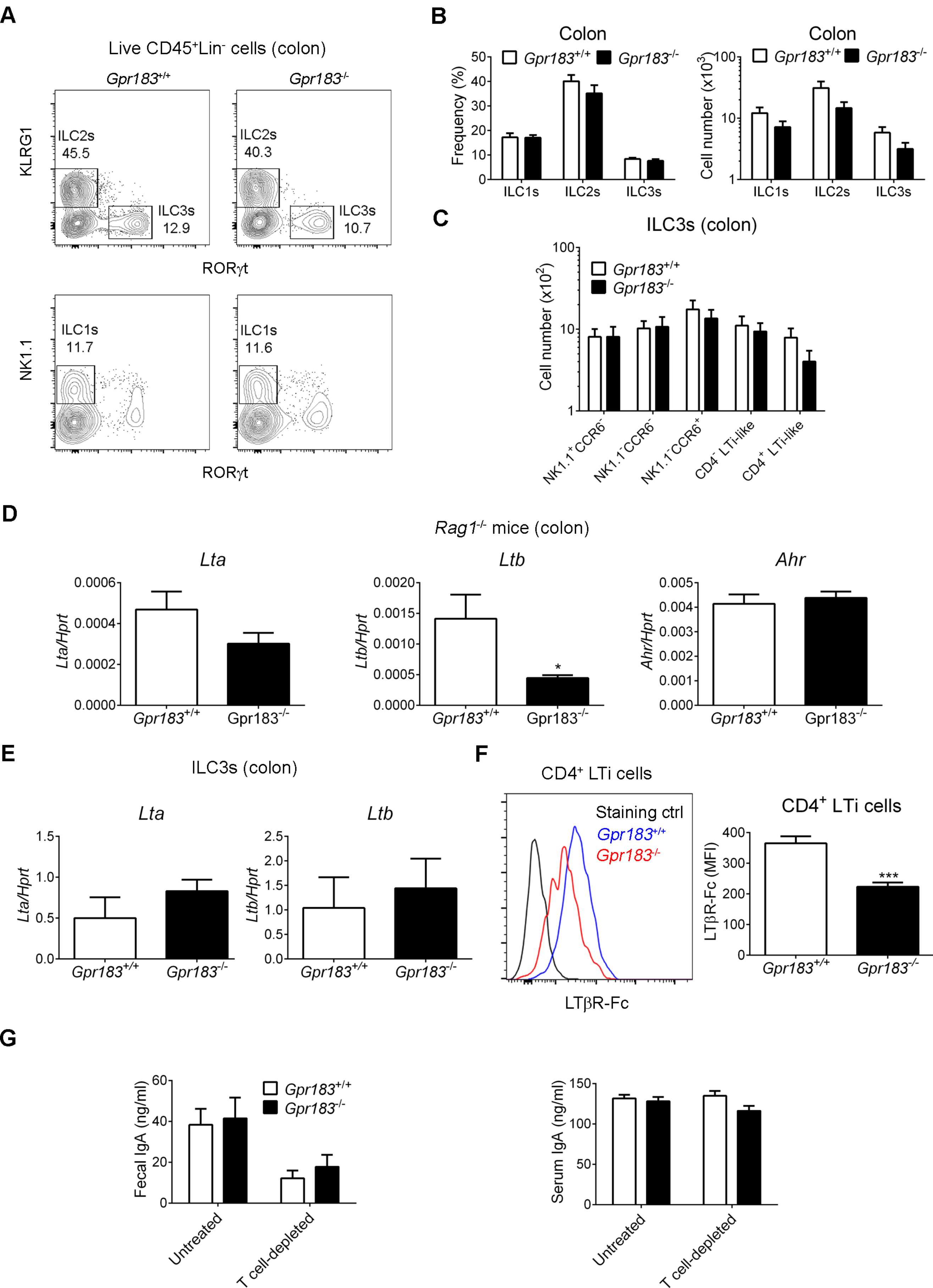

A
Live CD45+Lin- cells (colon)
Gpr183+/+
Gpr183-/-
KLRG1
ILC2s 45.5
ILC3s 12.9
ILC2s 40.3
ILC3s 10.7
RORγt
NK1.1
ILC1s 11.7
ILC1s 11.6
RORγt
B
Colon
Frequency (%)
Cell number (x10³)
ILC1s
ILC2s
ILC3s
C
ILC3s (colon)
Cell number (x10²)
NK1.1+CCR6-
NK1.1-CCR6-
NK1.1-CCR6+
CD4- LTi-like
CD4+ LTi-like
D
Rag1-/- mice (colon)
Lta
Ltb
Ahr
Lta/Hprt
Ltb/Hprt
Ahr/Hprt
E
ILC3s (colon)
Lta
Ltb
F
CD4+ LTi cells
Staining ctrl
LTβR-Fc
LTβR-Fc (MFI)
G
Fecal IgA (ng/ml)
Serum IgA (ng/ml)
Untreated
T cell-depleted

Figure S6, related to Figure 4: ILC homeostasis, lymphotoxin expression, and IgA production in *Gpr183*-deficient mice.

(A and B) Frequency and number of ILC subsets in the colon (incl. cecum) of *Gpr183*$^{+/+}$ and *Gpr183*$^{-/-}$ mice as determined by flow cytometry (*n* =13-15). After gating on live CD45^{+}Lin^{-} single lymphocytes, ILC1s/NK cells were gated as NK1.1^{+}RORγt^{-} cells, ILC2s as KLRG1^{+}RORγt^{-} cells, and ILC3s as RORγt^{+}KLRG1^{-} cells. (C) Number of ILC3 subsets in the colon (incl. cecum) of *Gpr183*$^{+/+}$ and *Gpr183*$^{-/-}$ mice was determined by flow cytometry (*n* =9-10). (D) *Lta, Ltb*, and *Ahr* mRNA expression in the colon of *Gpr183*$^{+/+}$ and *Gpr183*$^{-/-}$ mice on a *Rag1*-deficient background (*n* =8-15). mRNA expression was normalized to *Hprt*. (E) *Lta* and *Ltb* mRNA expression in sorted colonic ILC3s from *Gpr183*$^{+/+}$ and *Gpr183*$^{-/-}$ mice on a *Rag1*-deficient background (*n* =2). (F) $LT\alpha_1\beta_2$ surface expression on *Gpr183*$^{+/+}$ and *Gpr183*$^{-/-}$ CD4^{+} LTi-like ILC3s from mesenteric lymph nodes was detected by staining with LTβR-Fc fusion protein (*n* =5). Black histogram represents background staining (secondary Ab and streptavidin only). (G) IgA concentrations in serum and feces of *Gpr183*$^{+/+}$ and *Gpr183*$^{-/-}$ mice (*n* =5-13). Mice were either untreated or received α-TCRβ Ab to deplete T cells. Data are represented as mean ± SEM. *, P <0.05; ***, P <0.001 by Student's *t* test. Data are representative of or combined from two (E-G), three (D), five (C), or seven (A, B) experiments.

Figure S7

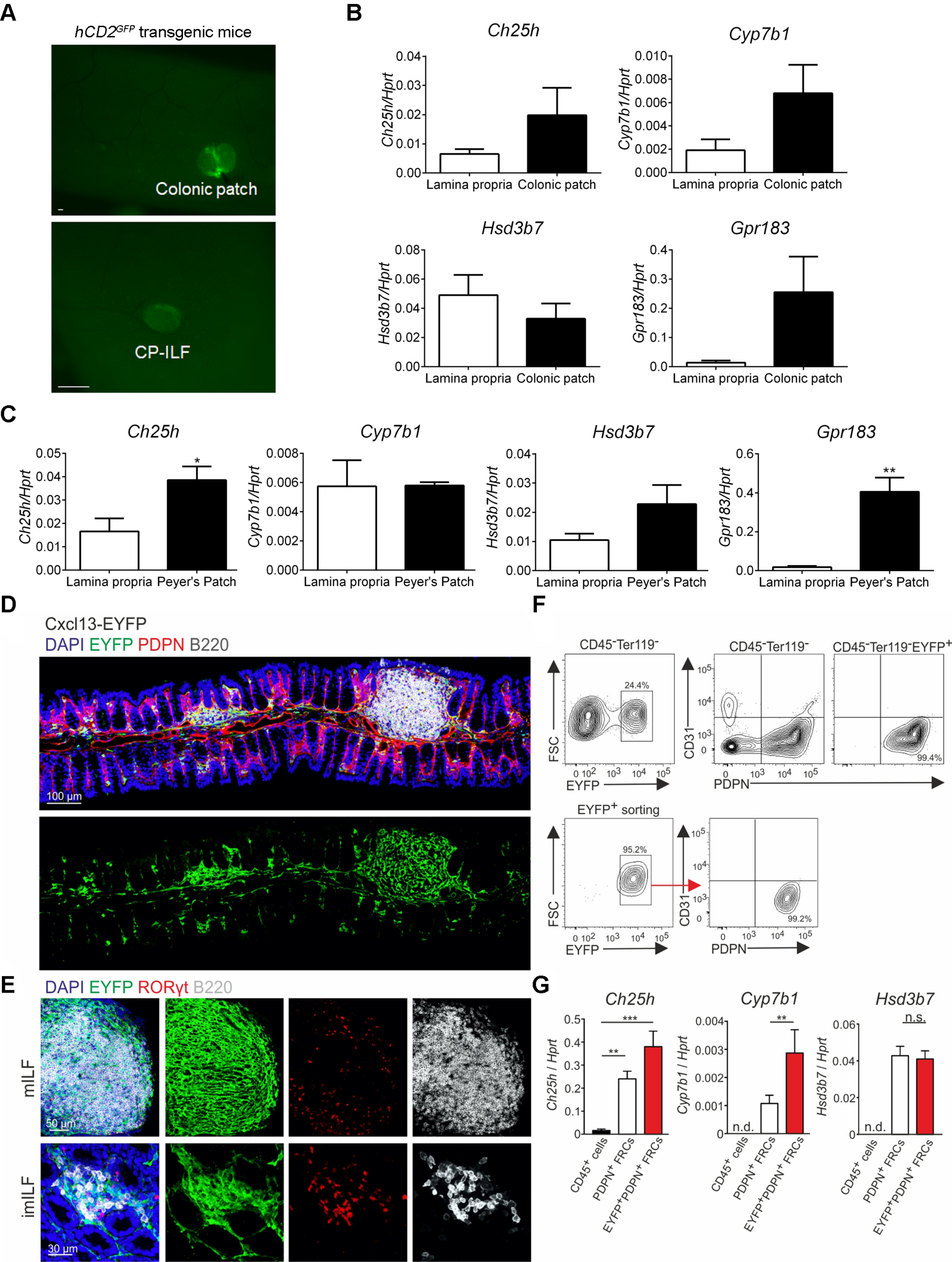

A
hCD2^GFP transgenic mice
Colonic patch
CP-ILF
B
Ch25h
Ch25h/Hprt
Lamina propria
Colonic patch
Cyp7b1
Cyp7b1/Hprt
Hsd3b7
Hsd3b7/Hprt
Gpr183
Gpr183/Hprt
C
Ch25h
Ch25h/Hprt
Lamina propria
Peyer's Patch
Cyp7b1
Cyp7b1/Hprt
Hsd3b7
Hsd3b7/Hprt
Gpr183
Gpr183/Hprt
D
Cxcl13-EYFP
DAPI EYFP PDPN B220
100 μm
E
DAPI EYFP RORγt B220
mILF
50 μm
imILF
30 μm
F
CD45⁻Ter119⁻
24.4%
FSC
EYFP
CD31
PDPN
CD45⁻Ter119⁻EYFP⁺
99.4%
EYFP⁺ sorting
95.2%
99.2%
G
Ch25h
Ch25h / Hprt
Cyp7b1
Cyp7b1 / Hprt
Hsd3b7
Hsd3b7 / Hprt
n.d.
n.s.
CD45⁺ cells
PDPN⁺ FRCs
EYFP⁺PDPN⁺ FRCs

Figure S7, related to Figure 5 and 6: Expression of GPR183 ligand-generating enzymes in colonic patches, Peyer's Patches, and colonic CXCL13^{+} stromal cells.

(A) Microdissection of colonic lymphoid tissues. Colons were harvested from h*CD2*^{GFP} transgenic mice and GFP^{+} lymphoid structures (colonic patch, CP-ILF) were visualized using a fluorescence stereomicroscope. Scale bars (white) are 200 μm. (B) *Ch25h*, *Cyp7b1*, *Hsd3b7*, and *Gpr183* mRNA expression in micro-dissected colonic patches from h*CD2*^{GFP} transgenic mice was compared to lamina propria (*n* =6). (C) *Ch25h*, *Cyp7b1*, *Hsd3b7*, and *Gpr183* mRNA expression in Peyer's Patches from human *CD2*^{GFP} transgenic mice was compared to micro-dissected lamina propria (*n* =4). (D) Immunofluorescence microscopy of colon from *Cxcl13*-EYFP reporter mice. EYFP^{+} cells (green), Podoplanin (PDPN)$^{+}$ cells (red), and B cells (B220^{+}, white) are shown. Nuclei were visualized with DAPI (blue). Scale bar (white) is 100 μm. (E) Localization of CXCL13-EYFP^{+} cells in colonic ILFs. Immature (imILF) and mature (mILF) ILF are shown. Scale bar (white) is 30 μm. (F) Phenotype and sorting of CXCL13-EYFP^{+} cells from the colon of *Cxcl13*-EYFP reporter mice. (G) *Ch25h*, *Cyp7b1*, and *Hsd3b7* mRNA expression in sorted CD45^{+} cells, PDPN^{+} fibroblastic reticular cells (FRCs), and CXCL13-EYFP^{+} PDPN^{+} FRCs from the colon (*n* =5). mRNA expression was normalized to *Hprt*. n.d., not detectable; n.s., not significant. Data are represented as mean ± SEM. *, P <0.05; **, P <0.01; ***, P <0.001 by Student's t test (C) or one-way ANOVA. Data are representative of or combined from one (C, F, G) or two (B, D, E) experiments.
